# Simple Fluorescent Sensors Engineered with Catalytic DNA ‘MgZ’ Based on a Non-Classic Allosteric Design

**DOI:** 10.1371/journal.pone.0001224

**Published:** 2007-11-21

**Authors:** William Chiuman, Yingfu Li

**Affiliations:** Department of Biochemistry and Biomedical Sciences, McMaster University, Hamilton, Ontario, Canada; Vanderbilt University, United States of America

## Abstract

Most NAE (nucleic acid enzyme) sensors are composed of an RNA-cleaving catalytic motif and an aptameric receptor. They operate by activating or repressing the catalytic activity of a relevant NAE through the conformational change in the aptamer upon target binding. To transduce a molecular recognition event to a fluorescence signal, a fluorophore-quencher pair is attached to opposite ends of the RNA substrate such that when the NAE cleaves the substrate, an increased level of fluorescence can be generated. However, almost all NAE sensors to date harbor either NAEs that cannot accommodate a fluorophore-quencher pair near the cleavage site or those that can accept such a modification but require divalent transition metal ions for catalysis. Therefore, the signaling magnitude and the versatility of current NAE sensors might not suffice for analytical and biological applications. Here we report an RNA-cleaving DNA enzyme, termed ‘MgZ’, which depends on Mg^2+^ for its activity and can accommodate bulky dye moieties next to the cleavage site. MgZ was created by in vitro selection. The selection scheme entailed acidic buffering and ethanol-based reaction stoppage to remove selfish DNAs. Characterization of MgZ revealed a three-way junction structure, a cleavage rate of 1 min^−1^, and 26-fold fluorescence enhancement. Two ligand-responsive NAE sensors were rationally designed by linking an aptamer sequence to the substrate of MgZ. In the absence of the target, the aptamer-linked substrate is locked into a conformation that prohibits MgZ from accessing the substrate. In the presence of the target, the aptamer releases the substrate, which induces MgZ-mediated RNA cleavage. The discovery of MgZ and the introduction of the above NAE sensor design strategy should facilitate future efforts in sensor engineering.

## Introduction

NAE (nucleic acid enzyme) sensors have two essential components: a receptor and a transducer. Similar to protein enzymes that are allosterically regulated by the presence of intra- or extracellular signals, an NAE sensor (also referred to as allosteric NAE) operates by activating or suppressing the catalytic activity of the NAE when the receptor recognizes its target. For NAEs that require a cofactor or an environmental cue to perform catalysis, they can be directly utilized for sensing applications as they already contain an inherent receptor. However, to detect a substance other than the catalytic constraint, an external receptor must be introduced.

Aptamers are single-stranded DNA or RNA molecules that can selectively bind to a specific ligand with high affinity and specificity. They can be routinely isolated from random sequence libraries through in vitro selection [1], [2] or SELEX (Systematic Evolution of Ligands by EXponential enrichment) [3]. The SELEX technique has been applied to a wide variety of targets ranging from small organic compounds to proteins to whole cells [4], [5]. With such target diversity, aptamers are regarded as a new class of receptors that can rival antibodies [6]. Aptamers (particularly DNA aptamers) offer several features that are attractive for biosensor development, which include high tolerance to denaturation, high chemical stability, ease of synthesis and manipulation, and low cost. However, aptamers alone usually cannot function as sensors because they do not posses an inherent ability to report their binding activities.

There are several strategies that can transduce the ligand-binding event of an aptamer to a physically detectable signal, such as change in fluorescence. These include aptamer beacon [7], excimer aptamer [8], modular aptameric sensor [9], signaling aptamer [10], structure-switching aptamer [11], fluorescence polarization [12] and allosteric NAE [13]–[25]. We chose allosteric NAE as the basis of our sensor development program simply because NAE is able to convert one binding event to multiple signals during catalytic turnovers, and it could be utilized as a platform to conduct aptamer selection [26]–[32]. The enzymatic domains of allosteric NAEs reported to date are mostly RNA-cleaving ribozymes found in nature [33] or RNA-cleaving DNA enzymes (DNAzymes or deoxyribozymes) artificially created by in vitro selection [34]. There are three main reasons why RNA-cleaving systems are usually favored over NAEs that catalyze other chemical reactions: (1) RNA-cleaving NAEs have been exhaustively studied because of their high occurrence in nature and their potential application in gene therapeutics. As a consequence, there are many valuable data that can facilitate biosensor engineering; (2) the progress of RNA cleavage can be easily monitored, as a typical RNA cleavage reaction produces two shorter oligonucleotide fragments; (3) the catalyzed rates of RNA cleavage are among the fastest of all reactions catalyzed by NAEs.

To synchronize cleavage activity with a change in fluorescence, molecular engineers typically attach a fluorophore and a quencher to opposite ends of an RNA substrate such that when the substrate is cleaved, the fluorophore and the quencher are physically separated from each other, with a concomitant increase in fluorescent intensity. This specific dye arrangement usually yields less than 10-fold fluorescence enhancement [35]–[38]. It is conceivable that the signaling magnitude can be greatly improved by simply moving the fluorophore and the quencher closer to the cleavage site, given that the efficiency of resonance energy transfer between any two dyes is inversely proportional to the sixth power of the separating distance [39]. Nonetheless, such operation might make an NAE incompetent at cleaving its substrate for the following reasons: (1) most RNA-cleaving NAEs were not intentionally selected to cleave substrates that have dyes at the cleavage site; (2) dye-bulkiness might sterically hinder the enzyme to access the cleavage site; (3) considering that most dyes also contain chemically functional moieties, the interactions between the scissile ribo-linkage and the enzyme might be disrupted due to the formation of an alternative interaction network.

**Figure 1:**
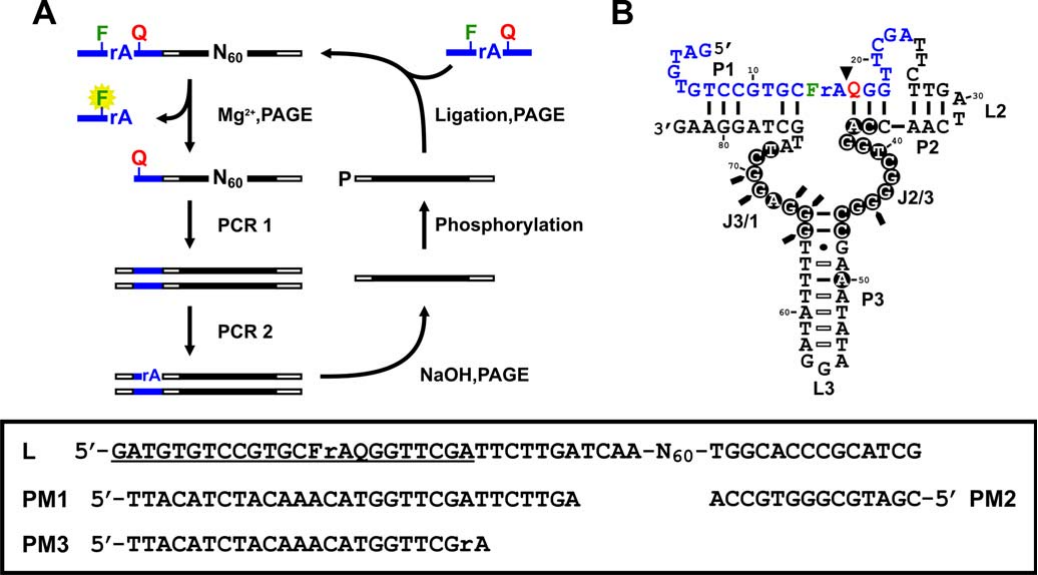
(A) In vitro selection scheme. The self-cleaving construct of the library consists of a DNA substrate (blue bars), 2 primer binding sites (open bars) and a random library (black bars). The substrate has one ribonucleotide, rA, flanked immediately by a fluorescein (F)- and a DABCYL (Q)-modified deoxyribothymidine. DNA species that are able to self-cleave in the presence of Mg^2+^ are isolated by denaturing PAGE and subsequently subjected to PCR- amplification. A ribo-linkage (rA) is introduced to the top strand of the PCR product during PCR 2, which enables the recovery of the DNAzyme strand after NaOH treatment followed by PAGE purification. To regenerate the self-cleaving construct, the DNA molecules are phosphorylated at the 5′ ends and ligated to the substrates. The selection cycle continues until a desired cleavage activity is reached. N represents A, G, C or T; P, phosphate. (B) MgZ in cis. Filled circles, absolutely conserved residues; Open bars, covariations; Filled triangle, cleavage site; Filled arrows, DMS methylation interfered residues; F, fluorescein-dT; Q, DABCYL-dT. Inset: random library in the self-cleaving construct (L) and the primers used (PM1-3). Substrate sequence is underlined.

Previous efforts from our group have led to the report of at least 9 DNAzymes that could efficiently cleave a chimeric DNA/RNA substrate that contains a lone RNA linkage (rA, adenine ribonucleotide) sandwiched between two deoxyribothymidines modified with a fluorescein (F) and a DABCYL (Q; 4-(4-dimethylaminophenylazo) benzoic acid), respectively (Figure 1 inset, underlined) [40]–[43]. These DNAzymes were isolated by in vitro selection and they were anticipated to yield a large fluorescence increase upon substrate cleavage (owing to the close proximity of F and Q on the uncleaved substrate). However, due to the fact that divalent transition metal ions (such as Mn^2+^, Cd^2+^, Co^2+^, Ni^2+^) are required for the DNAzyme function and that these metal ions have been found to be strong fluorescence quenchers [44], the best signal enhancement attained was only ∼3-fold better than that of the end-to-end dye arrangement described above. The necessity of transition metal ions also hinders the recruitment of these DNAzymes in biological applications because the metal toxicity could become a concern. We were intrigued, however, by the fact that none of these DNAzymes recruited Mg^2+^ as a cofactor, even though Mg^2+^ was included in the selection buffer. To investigate whether it is a chemical challenge for DNA to catalyze the cleavage of the same substrate in the presence of Mg^2+^, we set out to carry out an in vitro selection study to isolate similar DNAzymes when Mg^2+^ was given as the only divalent metal ion in the reaction mixture.

## Results

### In vitro selection

The starting library contained, in the 5′ to 3′ direction, the substrate, the 5′ primer-binding site, a 60-nucleotide random region (N_60_) and the 3′ primer-binding site (Figure 1A). The random region in one-third of the library was composed of equal ratio of A, G, C and T at every position, while the random region of the remaining library was composed of the sequences of the known ‘OA’ DNAzymes with a degeneracy of 0.3 (70% wild type nucleotide and 10% each of the other nucleotides for every nucleotide position within the random region). OAs are six RNA-cleaving DNAzymes previously isolated by our group and all of them use divalent transition metal ions as cofactors [42]. They were included in the starting pool because we speculated that some OA variants might be able to use Mg^2+^ as well. To isolate Mg^2+^-dependent DNAzymes, the DNA molecules with the covalently attached substrate were allowed to self-cleave with 10 mM Mg^2+^ at pH 7.5 for 4 hours. Cleaved sequences were isolated by denaturing PAGE and PCR-amplified with primers PM1 and PM2 (Figure 1 inset). A tiny amount of the PCR product was used to seed the second PCR where a ribo-linkage was introduced to the sense strand by using a ribo-terminated primer (PM3). After boiling the second PCR product in NaOH, the ribo-linkage was hydrolyzed, and as a result, three oligonucleotides of unequal lengths were produced. The DNAzyme strand was purified by denaturing PAGE, phosphorylated at the 5′, and ligated to the substrate. Such assembled DNA pool was then used for the next round of selection. Another 11 cycles were performed with the reaction time progressively reduced from 4 h to 6 s for the purpose of enriching the most efficient DNA sequences (see Materials and Methods for the reaction time allowed for each cycle). The final generation (G12) was cloned and sequenced.

Twenty-one sequences were obtained (Figure S1) and they are termed G12SD-X, where X is the clone number. Two sequences, G12SD-1 and G12SD-2, from the two dominating sequence classes were arbitrarily chosen for further examination. To our surprise, both DNA species were able to cleave the RNA linkage in the absence of Mg^2+^. However, the best cleavage yields in any cases were less than 10% (data not shown). We then attempted to optimize G12SD-1 and G12SD-2 through reselection (i.e., in vitro selection using degenerate libraries). Without much success, the optimized species only showed a small increase in self-cleavage yield (≤10%) and no improvement in intermolecular cleavage (Figure S2, only the optimized G12SD-1 is shown).

As we later found out that the activity of G12SD-1 could be severely suppressed in low pH conditions, several procedures in the selection scheme were modified, especially after the substrates were ligated to the DNAzymes. The changes made include: (1) running denaturing PAGE in 1× TME solution (30 mM Tris, 54 mM MES, 1 mM Na_2_EDTA, pH 6.2) instead of 1× TBE (89 mM Tris, 89 mM boric acid, 1 mM EDTA, pH 8.3); (2) eluting DNA from the gel in 10 mM MES (pH 5.5) rather than in the solution containing 10 mM Tris-HCl (pH 7.5), 200 mM NaCl and 1 mM EDTA; (3) reducing the elution time from 30 min to 10 min; (4) stopping the reaction by adding 2.5× equivalent volume of 100% ethanol. Using G5 (where a strong cleavage activity was first observed) as our starting pool, a new selection experiment was conducted with the modified selection scheme, in hope of isolating DNAzymes that are Mg^2+^-dependent and more efficient than G12SD. The reaction was restricted to 1 min in the first round of selection and progressively shortened to 10 s over the next 6 rounds (see Materials and Methods for the reaction time allowed for each cycle). Four sequence classes in the final generation were found to use Mg^2+^ for substrate cleavage (Figure S1); sequences that perform Mg^2+^-dependent RNA cleavage are termed MgZ-X. One sequence from each class was arbitrarily chosen, and their trans-cleavage activities were assessed. The two sequences (MgZ-5 and MgZ-7) that showed higher activities were subjected to reselection. Given that the reselected MgZ-5 showed higher cleavage activity than the reselected MgZ-7, one of the MgZ-5 variants (56 in total) was selected for the remaining study. It is referred to as ‘MgZ’ for convenience (Figure 1B).

### Identification of catalytic motifs

According to our proposed secondary structure model (Figure 1B), MgZ adopts a three-way junction structure when binding to the substrate. Stems P1 and P2 are formed by the hybridization between the substrate (blue) and the two binding arms located at the 5′ and the 3′ ends of the DNAzyme (black), thus creating a bulge at the cleavage site. Stem P3 is formed on the DNAzyme strand, and it interlinks P1 and P2 through two stretches of 8 nucleotides, namely J2/3 and J3/1 (J2/3 represents the junction between P2 and P3; similar nomenclature applies to J3/1). Sequence analysis of the reselected clones revealed two observations: (1) the existence of P3 is strongly supported by the covariable nucleotide pairs found in the region between G_48_ and T_64_ (highlighted with open bars); (2) the highly conserved nucleotides (black circles) are concentrated mostly in J2/3 and J3/1. Since some of these nucleotides are also prone to dimethyl sulfate (DMS) methylation interference (highlighted with filled arrows), it is reasonable to assume that J2/3, J3/1 and the open end of P3 harbor the catalytically essential nucleotides or presumably, the catalytic core of MgZ. For details in the nucleotide conservation patterns and the DMS methylation interference result, see Figures S3 and S4.

To validate the proposed secondary structure, MgZ was first converted to the trans format by disjoining the DNAzyme strand from the substrate (Figure 1B). The 3′ tail of the substrate was then extended with 5′-TTCTTGATCAA-3′ such that a stronger P2 could be formed (Figure S5). Based on these constructs, a series of substrate and MgZ variants were synthesized and tested to assess the catalytic importance of P1, P2 and P3. In brief, all 3 stems are required for cleavage activity. Deletion of either DNA strand of any stem or incorporation of several mismatches within the stem motifs is detrimental to catalytic function. P1, P2 and P3 can be shortened to 6, 4 and 6 base pairs, respectively without a drop in cleavage yield. The sequence contexts of P1 and P2 can be altered as long as the Watson-Crick base pairs are retained. However, the catalytic activity would be compromised to different extents, depending on the modifications. P3 can be covaried with no effect on the cleavage activity, except the two C≡G base pairs and the following G•T wobble pair at the open end (Figure 1B). The sequence context of loop L3 is not important for catalysis. Details of individual mutant constructs and experimental results are presented in Figures S5, S6, S7 and S8.

### Signaling property of the DNA enzyme

**Figure 2:**
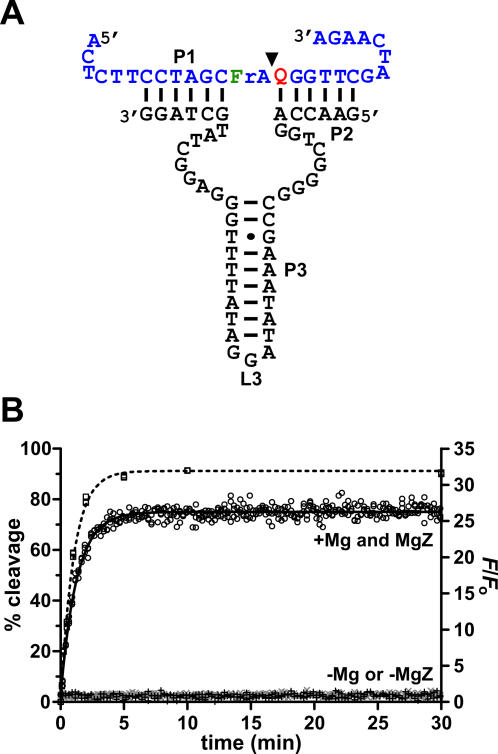
(A) MgZ in trans. Shown here is a specific combination of substrate and MgZ variant that generates the largest signal enhancement upon substrate cleavage. (B) Kinetic analyses. □ refers to the left y-axis (% cleavage); ○, +(−Mg) and×(−MgZ) refer to the right y-axis (*F*/*F*_o_). The % cleavage vs. time data were fitted to *Y* = *Y*_f_(1–e^−*k*_c_^ *^t^*). Fitting-curve is shown as ---. *Y*, cleavage yield; *Y*_f_, final cleavage yield; *k*_c_, observed cleavage rate. The *F*/*F*_o_ vs. time data were fitted to *F*/*F*_o_ = initial *F*/*F*_o_+(final *F*/*F*_o_−initial *F*/*F*_o_)×(1−e^−*k*_s_^ *^t^*); initial *F*/*F*_o_ = 1.0. Fitting curve is shown as —. *F*/*F*_o_, fluorescence enhancement; *k*_s_, signal-enhancement rate. Cleavage rate = 1 min^−1^ with Y_f_ = 91%. Signaling rate = 0.8-fold min^−1^ with final *F*/*F*_o_ = 26-fold. Reaction condition: 5 nM substrate, 1 µM DNAzyme, 70 mM HEPES (pH 8.0), 40 mM MgCl_2_, 0.001% Tween-20, 30°C.

Radioactive and fluorescence-monitored cleavage assays were carried out in a single-turnover condition with several combinations of substrate constructs and MgZ variants that had been used to characterize the structure of MgZ (see Figure S9 for results). The combination that gave the best signaling performance is shown in Figure 2A. Its cleavage and fluorescence kinetics are plotted in Figure 2B. Cleavage yields over time (γ) refer to the left y-axis (% cleavage yield); other data points refer to the right y-axis (*F*/*F*_o_, fluorescence enhancement). The cleavage reaction was initiated by adding excess DNAzymes to the substrate in 70 mM HEPES (pH 8.0), 40 mM MgCl_2_ and 0.001% Tween-20 at 30°C (the optimal condition with pH≤8.0). More fluorescence was generated as the reaction proceeded. Since the level of fluorescence enhancement increased at a similar rate as that of the cleavage yield, the release of cleavage fragments (at least one of the two) from the DNAzyme was not a limiting step for fluorescence development. The cleavage rate of the DNAzyme was 1 min^−1^ with 91% final cleavage yield and the signaling rate was 0.8-fold min^−1^ with 26-fold maximum signal enhancement. It should be noted that the observed fluorescence increase was specific to the DNAzyme-catalyzed substrate cleavage, as no fluorescence enhancement was observed when either Mg^2+^ or MgZ was excluded from the reaction mixture (Figure 2B).

### Sensor engineering

There are a variety of engineering methods to assemble NAE sensors. The most well known method is to link a particular aptamer to an RNA-cleaving NAE (such as hammerhead ribozyme) through a communication module [14]. Communication module is usually a weak duplex scaffold that changes its base-pairing pattern, depending on whether the aptamer domain is in the target-bound state or the target-free state. Alteration in the base-pairing pattern, in turn, causes either activation or repression of the activity of NAE. In spite of many engineering approaches, the common theme in all of the existing NAE sensors is to put NAE under allosteric control [13]–[32]. Here we used an alternative approach to design an ATP sensor and an ADP sensor using two existing aptamers and MgZ. The purposes of these experiments were mainly two-fold: (1) to show the utility of MgZ in sensor development; (2) to demonstrate that NAE sensors can also be engineered on the basis of a mechanism that regulates the accessibility of the substrate through ligand binding. For convenience, we term our strategy ‘non-classic allosteric design’.

**Figure 3:**
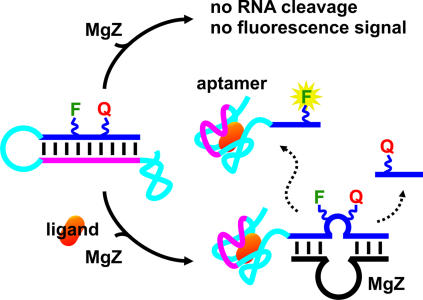
Sensor design and mechanism. Blue, substrate; cyan, aptamer; magenta, antisense element. See text for details.

The sensor design and mechanism are depicted in Figure 3 and described as follows. An aptamer (cyan) is engineered to harbor an antisense element (magenta) in its variable region. By conjugating such aptamer to the fluorogenic substrate of MgZ, the substrate is sequestered in an extended conformation (such as a duplex) by hybridization with the antisense element. With this arrangement, MgZ has no access to the substrate and no cleavage occurs. In the presence of the target, however, the aptamer pulls away the antisense element from the substrate and folds into the target-bound conformation. Consequently, MgZ is able to bind and cleave the substrate. Due to the physical separation of F and Q, higher level of fluorescence will be generated in response to the presence of target.

### ATP sensor

The receptor of the ATP sensor is an ATP-binding DNA aptamer that was previously isolated by the Szostak group [45]. It has high affinity to ATP and its analogs such as adenosine and AMP; however, the aptamer does not bind to other nucleotides. The NMR model of the AMP-aptamer complex reveals that the aptamer adopts a stem-loop structure with two binding sites [46]. Given that the loop is not involved directly in target binding, it poses a good site to incorporate an antisense element. A series of aptamer-antisense sequences were designed and annealed to the 5′ or the 3′ ends of the substrates. These constructs were screened by radioactive cleavage assay for the ability to block the substrate from being cleaved by MgZ in the absence of ATP, as well as the plasticity to switch from the substrate-blocking conformation (closed) to the target-bound conformation (open), which in essence allows MgZ to bind and cleave the substrate, when ATP is added.

**Figure 4:**
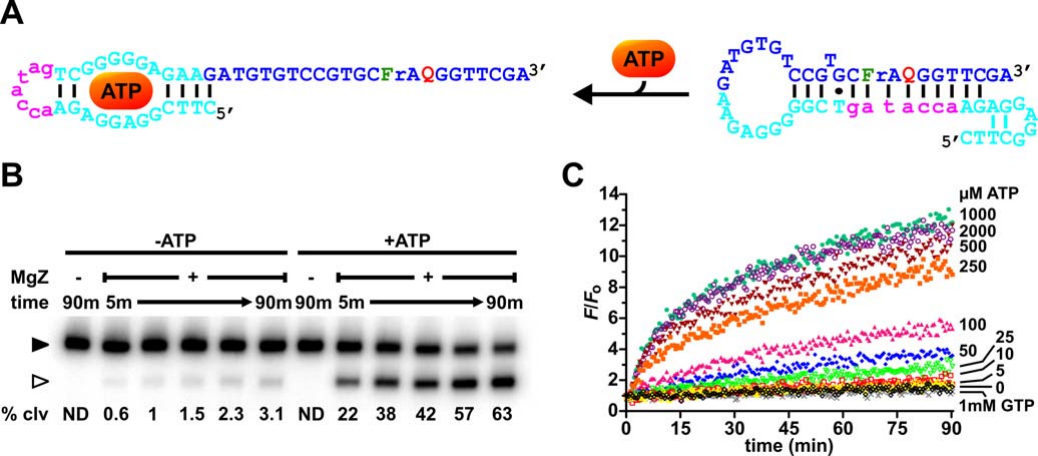
ATP sensor. (A) ATP-ASAP. (B) Phosphorimage of the radioactive cleavage assays. Cleavage reactions were carried out with 5 nM ATP-ASAP, 50 nM MgZ and 1 mM ATP (in the case of ATP-induced cleavage) in 50 mM HEPES (pH 7.0), 20 mM MgCl_2_, 0.001% Tween-20 at room temperature. m, minutes; % clv, % cleavage; ND, not detectable; ▸, substrate; ▹, 5′ cleavage fragment. (C) Fluorescence kinetics in response to various concentrations of ligands. Similar reaction conditions in panel B were applied.

One of the constructs, which showed the largest increase in cleavage yield over the background, has a 7-nucleotide antisense element that, in conjunction with the flanking nucleotides of the aptamer, locks the substrate in a 13-base pair bulged duplex (Figure 4A; same color scheme as in Figure 3). Although minor cleavage by MgZ still occurred with this scaffold (Figure 4B), the amount of cleavage products was elevated by at least 20-fold (within the 90 min time frame) in response to 1 mM ATP. This observation clearly indicates that substrate cleavage was triggered only through the recognition of the target and not by means of random events. To assess the signaling performance of the sensor, fluorescence enhancement kinetics correlated to various concentrations of ATP were surveyed. As shown in Figure 4C, higher rate of fluorescence increase and larger fluorescence enhancement correspond to higher concentration of ATP up to 1 mM (a smaller signal enhancement with 2 mM was attributable to the pH effect of ATP; see Figure S10). The sensor has a detection limit of 5–10 µM and a greater sensitivity in the range of 10–250 µM. Similar to the “0 µM ATP” kinetics (black), the signal enhancement with 1 mM GTP (grey) was also negligible over time, which reiterates the fact that the aptamer (therefore, the sensor) is selective for ATP.

**Figure 5:**
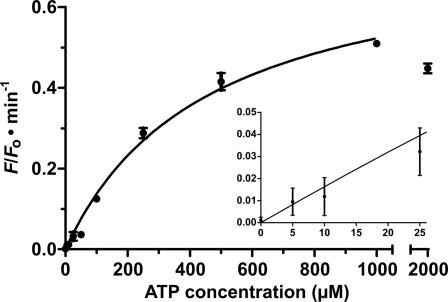
Initial rate of fluorescence enhancement vs. ATP concentration. Fitting curve: *v*_o_ = (*v*_max_×[ligand])/(*K*_d_+[ligand]). *v*_o_, initial rate of signal enhancement (see Materials and Methods); *v*_max_, maximal rate of signal enhancement; *K*_d_, dissociation constant. Error bar represents the standard deviation of three independent assays. *v*_o_ with 2 mM ATP was omitted in the curve fitting process due to substantial fluorescence quenching by high [ATP] (Figure S10). Inset: *v*_o_ vs. low [ATP].

In comparison with the unmodified substrate (Figure 2), the assemblage of antisense element, substrate and aptamer into a tripartite structure-switching substrate (termed “ASAP”) did not allow RNA cleavage to occur with a similar rate, cleavage yield or fluorescence enhancement even under the ATP-saturating condition (Figures 4B and 4C). There are at least three plausible reasons: (1) the reaction was not conducted in the optimal condition for substrate cleavage (Figure 2 legend) but in a condition specifically optimized for ATP-induced cleavage (Figure 4 legend); (2) the ASAP complexes might have folded into alternative structures that prohibited conformational transition and substrate access; (3) the closed conformation is strongly favored over the open conformation. To determine if the binding affinity of the aptamer was deteriorated in the context of ASAP, the initial rates of fluorescence enhancement were calculated and plotted against the ATP concentrations (Figure 5). Curve-fitting with the one site binding model revealed an apparent *K*_d_ of 456±47 µM, which is significantly higher than that of the aptamer alone (∼6 µM) [45]. Since an increase in the apparent *K*_d_ was also observed in several allosteric NAEs [16], [18], [27], [28], it might implicate that an extra free energy is usually required to drive the conformational transition in both allosteric and ASAP systems. Based on the initial rates of fluorescence enhancement, the sensor was found to have >445-fold higher selectivity for ATP over GTP. It is important to note that the data in Figure 5 could only be fitted with the one site binding model, despite the NMR structure reveals two binding sites [46] and previous studies have suggested that the aptamer binds cooperatively to 2 ATP molecules [10], [16]. This discrepancy might be due to that the transition of ASAP from the closed to the open conformation can only be induced through the binding of 2 ATP molecules at the same time. Such a change in the binding mode relative to that of the parent aptamer might also contribute to the apparent decrease in binding affinity. Regardless, the sensor described here is comparable to most DNAzyme-based [16] and even aptamer-based ATP sensors [9]–[11] in terms of sensitivity, selectivity and the dynamic range of detection.

### ADP sensor

**Figure 6:**
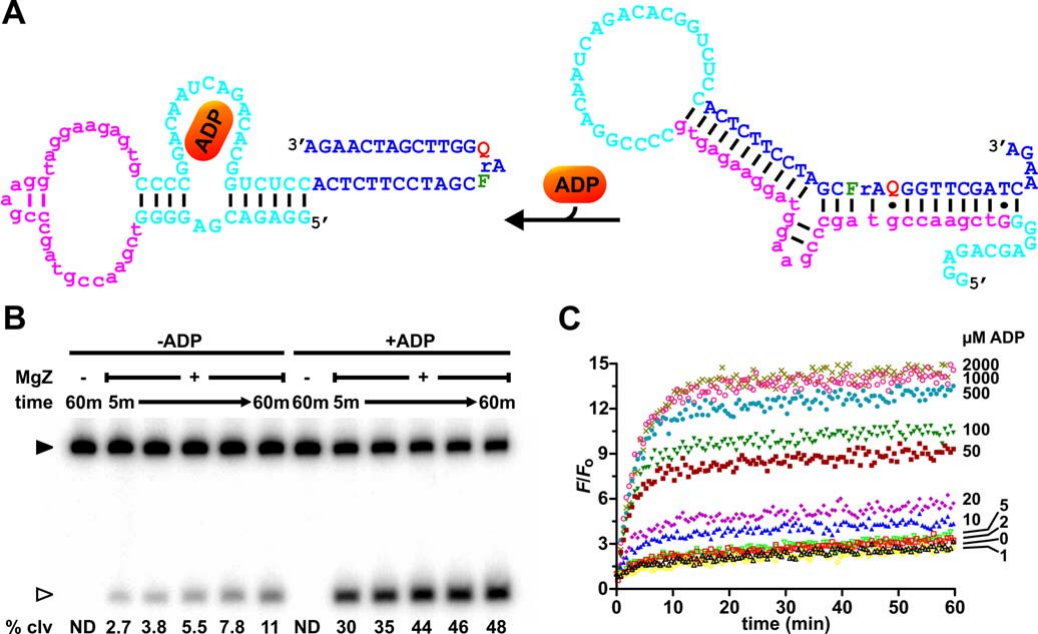
ADP sensor. (A) ADP-ASAP. (B) Phosphorimage of the radioactive cleavage assays. Cleavage reactions were carried out with 5 nM ADP-ASAP, 1 µM MgZ and 1 mM ADP (in the case of ADP-induced cleavage) in 50 mM HEPES (pH 7.0), 20 mM MgCl_2_, 0.001% Tween-20 at room temperature. ▸, substrate; ▹, 3′ cleavage fragment. (C) Fluorescence kinetics in response to various ADP concentrations. Similar reaction conditions in panel B were applied.

The receptor of the ADP sensor is an ADP-RNA aptamer isolated by the Diener group [47]. The aptamer has ∼300-fold higher selectivity for ADP over ATP and low affinity to other nucleotides. The ADP aptamer was proposed to have a stem loop structure, in which the loop is not important for its function and thus is a good site to incorporate an antisense element. A series of antisense elements were screened for ADP-induced substrate cleavage. One of the best ASAPs, shown in Figure 6A, has a 30-nucleotide long antisense element that locks the substrate in a 3-way junction scaffold. By combining this ASAP with MgZ, an ADP sensor was created. It should be emphasized that the antisense element is composed entirely of DNA. Although it would be more convenient to synthesize an RNA antisense element along with the aptamer sequence in one piece, we have screened 10 different ASAP constructs that contain all RNA aptamer-antisense sequences, and none of them was able to inhibit substrate cleavage in the absence of the target, even with an antisense element that forms 14 base pairs with the substrate. A similar DNA counterpart, however, could show some substrate-blocking activity (data not shown).

**Figure 7:**
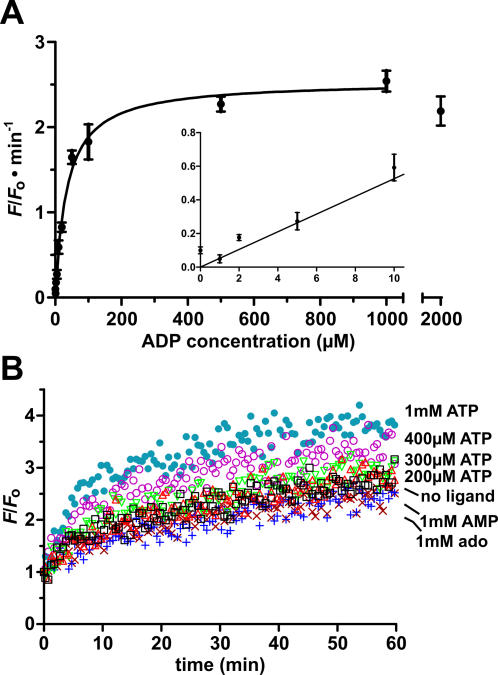
Sensitivity and specificity of ADP sensor. (A) Initial rate of fluorescence enhancement vs. ADP concentration. Fitting curve: *v*_o_ = (*v*_max_×[ligand])/(*K*_d_+[ligand]). Error bar: standard deviation of three independent assays. *v*_o_ with 2 mM ADP was omitted in the curve fitting process due to fluorescence quenching by high [ADP] (Figure S10). Inset: *v*_o_ vs. low [ADP]. (B) Fluorescence kinetics in response to non-cognate ligands. ado, adenosine; ×, 1 mM ado; +, 1 mM AMP; □, no ligand; ▵, 200 µM ATP; ▿, 300 µM ATP; ○, 400 µM ATP; •, 1 mM ATP.

To evaluate the sensor performance, both radioactive and fluorescent cleavage assays were performed. Phosphorimage of the radioactive cleavage assays (Figure 6B) shows that the sensor, in response to 1 mM ADP, had >10-fold increase in the cleavage yield over the background (no ADP) in the first 5 min. That difference, however, fell progressively to ∼4-fold over the next 55 min, as more ASAPs collapsed to alternative conformations that allowed substrate cleavage in the absence of the target whilst the ADP-induced cleavage went to near completion. Fluorescence enhancement kinetics correlated to various concentrations of ADP are plotted in Figure 6C. As indicated in the figure, higher ADP concentrations resulted in higher rate of fluorescence increase and larger fluorescence enhancement. The sensor has a low detection limit of 2 µM and a greater sensitivity in the range of 2–500 µM. The sensitivity is better resolved by plotting the initial rate of fluorescence enhancement against the ADP concentration, as shown in Figure 7A. By fitting the data from this plot into the one site binding model, an apparent *K*_d_ of 35±3 µM was revealed, which is higher than that of the aptamer alone (3 µM) [47]. This again suggests that more free energy is required for the aptamer to undergo conformational transition in the context of ASAP.

Shown in Figure 7B are the fluorescence enhancement kinetics corresponding to adenosine, AMP and ATP at various concentrations. In comparison with the background signal (no ligand), no extra fluorescence was generated with 1 mM adenosine, 1 mM AMP and 200 µM ATP. Nevertheless, with 300, 400 and 1000 µM ATP, larger fluorescence enhancements up to ∼3, 3.4 and 3.9-fold were detected within the 60 min time frame. Based on the initial rates of fluorescence enhancement corresponding to various ADP analogs at 1 mM, the sensor has >22-fold selectivity over adenosine and AMP, and 9-fold selectivity over ATP. However, at 200 µM of analyte, the sensor has >18-fold selectivity over ATP.

## Discussion

The key to the success of in vitro selection is to devise an effective selection method to separate the nucleic acid species with the function of interest apart from the inert species. However, even with a well-established selection protocol, some unwanted species with unexpected characteristics might still bypass the selection criteria and in our case, dominated the whole population. These molecules are identified as “selfish species” [48]. To eradicate these molecules in the course of selection, we first characterized them and then modified the selection scheme accordingly so as to reduce their chances of survival. Although our strategy was not perfect as some of them were still able to pass through, the pool distribution of Mg^2+^-utilizing DNAzymes must have grown much faster than the selfish species in consecutive selection cycles, and allowed the successful isolation of MgZ.

The discovery of MgZ has brought us to the conclusion that beside the normal RNA substrate and the chimeric DNA/RNA substrate [34], [49], the chemical challenge to cleave a DNA substrate with a crowded RNA linkage can also be overcome by catalytic DNAs assisted by Mg^2+^. However, it was intriguing to observe that MgZ also adopts a simple 3-way junction secondary structure that is common to all OA DNAzymes [42]. This prompted us to wonder why none of the sequenced clones (∼130) from our previous selection study recruited Mg^2+^ as the divalent metal ion cofactor [40], [42], if a similar structural framework was exploited. One possible reason is that the diversity of transition metal ion-dependent DNAzymes was significantly higher than that of the Mg^2+^-dependent DNAzymes in the initial library. Thus, the chances for the minor Mg^2+^-dependent species to survive the entire in vitro selection process would be small, as they could be easily lost. This notion could be further supported by a previous study where transition metal ion-promoted self-phosphorylating DNAzymes were found to be significantly more populated than the ones assisted by Mg^2+^ [50].

As discussed earlier, molecular engineers typically attach a pair of fluorophore and quencher at opposite ends of the substrate to couple the RNA-cleaving activity with fluorescence signals. To improve the signal enhancement attained by this end-to-end dye arrangement (<10-fold), the Lu group has added another quencher to one end of the NAE, which also positioned the quencher adjacent to the fluorophore for static quenching [51]. This new dye arrangement has been applied to two DNAzymes that are lead-dependent [52] and uranyl-specific [53], respectively, and the resulting constructs produced 10- and 16-fold signal enhancements upon substrate cleavage. Recently, we have studied the effects of the nature of dye moieties as well as their attaching positions along the substrate strand on the catalytic and signaling performance of an RNA-cleaving DNAzyme termed 8–17 [54]. 8–17 is very versatile in terms of its metal usage for catalysis (including Mg^2+^) [52], [55]–[57]. Although we learnt that 8–17 is able to accommodate dye compounds that are only one nucleotide away from the cleavage site and to generate ≤85-fold fluorescence enhancement, the cleavage activity will be severely impaired if Mn^2+^ is not supplemented in the reaction mixture. Clearly, the advent of new engineering methods described above have significantly advanced the development of better fluorogenic NAE platforms and eventually, NAE sensors. Nevertheless, these platforms might not be the ideal choices for engineers to apply in building sensors for biological applications. Here we took advantage of the in vitro selection technique and successfully isolated a Mg^2+^-dependent DNAzyme that could efficiently cleave a sterically hindered (and perhaps, chemically challenging) RNA linkage that is flanked immediately by fluorescein and DABCYL. Due to the close proximity of these two chromophores, substrate cleavage could lead to high fluorescence generation and in this case, 26-fold fluorescence enhancement above the background (substrate itself), which doubles the best fluorescence enhancement attained by the catalytic platforms previously isolated by the Li group [40]–[43]. Given that MgZ-catalyzed RNA cleavage went to almost completion in 5 min (Figure 2B), we believe MgZ will be a great signaling molecule to be exploited in sensor engineering for both analytical and biological applications.

To demonstrate how to build sensors using the MgZ system, we have chosen the ATP-binding DNA aptamer and the ADP-binding RNA aptamer as our model receptors. Using a new engineering approach, the aptamers were incorporated with antisense elements and annealed to the substrates to form ASAP complexes. In the absence of the target, ASAPs remained in the closed state; in the presence of the target, ASAPs switched to the open state and allowed MgZ to cleave the substrate, leading to high fluorescence generation. As depicted in Figure 3, ASAP is an isolated unit where both target recognition and conformational transition occur in chorus and independently from the DNAzyme. Since no modification is applied to the DNAzyme, its folding and catalytic activity would not be affected (presumably) to any extent when it binds to the substrate domain of ASAP, as the aptamer is attached to one extreme end of the substrate. Likewise, the aptamer should also not be disrupted by the folding of the DNAzyme through steric clashes. Therefore, the only constraint to build a similar type of sensor is to find a region of the aptamer that could be replaced by or incorporated with an antisense element. The classic allosteric engineering approaches, however, usually require the covalent attachment of the aptamer to NAE. Without NMR or crystal structures, it is very difficult to assemble them in such a way that steric clashes could be avoided. Although in vitro selection could be used to search for aptamer-NAE sequences that have the capacity to synchronize both functions and at the same time, maintain their full activities, there could be several outcomes based on the previous studies [14], [16], [26]–[32]: (1) allosteric NAEs that maintain the full activities of both aptamer and NAE; (2) only one domain maintains its full activity; (3) both activities are affected; (4) effective allosteric control is limited to suppression only; (5) poor allosteric NAEs due to high background signal; (6) no allosteric activity.

In comparison with the optimized NAE-based ATP and ADP sensors reported in the literature [16], [47], the sensors described in this study, which were created solely by rational design, have similar detection limit, detection range, specificity and selectivity. Given that the cleavage and fluorescence-signaling performance of MgZ were poorer when coupled with the ASAPs than with the unmodified substrate, the sensor performance might be further improved by screening a larger library of antisense elements via in vitro selection. It should be emphasized that in this study, we did not intend to optimize the sensors for practical applications but to illustrate how to incorporate aptamers in both DNA and RNA formats into the MgZ system. It is unclear, however, for what reasons that the all-RNA antisense elements we examined were not able to block the substrate from MgZ-catalyzed RNA cleavage, given that RNA-DNA hybridization, in general, has similar thermodynamic stability and instability to that of RNA-RNA duplex and DNA-DNA mismatches, respectively [58]. Currently, we are exploring the ASAP-MgZ system for aptamer selection. Further assessment of the ASAP design is thus warranted in the near future.

## Materials and Methods

### Chemicals and Oligonucleotides

DMS, piperidine, tetrabutyl-ammonium fluoride (TBAF) solution (1M in tetrahydrofuran), Tween-20 and nucleotide analogs including adenosine, AMP, ADP, ATP and GTP were obtained from Sigma-Aldrich. dNTPs were purchased from MBI Fermentas. [γ-^32^P]ATP and [α-^32^P]dGTP were from GE Healthcare Life Sciences or Perkin Elmer. All other chemicals were from BioShop (Burlington, Canada). Water was purified with a Milli-Q Synthesis A10 water purification system and then autoclaved. Standard and modified oligonucleotides were prepared by solid phase synthesis (Integrated DNA Technologies or Keck Biotechnology Resource Laboratory, Yale University). All oligonucleotides were purified by 8% denaturing PAGE before use. The 2′-OH protecting TOM (triisopropylsilyloxymethyl) group of the RNA linkage within the chimeric DNA/RNA substrate was removed by adding 500 µL of TBAF to ∼250 pmol of substrate which is in the form of a dry pellet, followed by overnight incubation at 60°C on a mixer. The reaction was quenched by adding 200 µL of H_2_O. The resulting product was precipitated with ethanol, resuspended in H_2_O, desalted on a Nanosep 3K device (Pall Life Sciences), and further purified by 8% denaturing PAGE.

### In vitro selection

The starting library consists of 500 pmol of random sequences in the context of 5′-TTCTTGATCAA-N_60_-TGGCACCCGCATCG-3′, where N is 25% A, 25% G, 25% C and 25% T, plus 165 pmol of each of the six OA degenerate libraries [42]. To prepare the self-cleaving DNA construct (L) shown in Figure 1 inset, the library was first ^32^P-labeled at the 5′-end in the presence of T4 PNK (T4 polynucleotide kinase; MBI Fermentas) and 10 µCi [γ-^32^P]ATP for 30 min. Non-radioactive ATP was then added to a final concentration of 1 mM. The mixture was further incubated for 30 min to ensure complete phosphorylation. The resulting product was ligated to the chimeric DNA/RNA substrate, 5′-GATGTGTCCGTGCFrAQGGTTCGA-3′, in the presence of an appropriate template and T4 DNA ligase (MBI Fermentas). The ligated products were purified by 8% denaturing PAGE and resuspended in H_2_O.

The library was heated at 90°C for 30 s and cooled to room temperature (∼23°C) for ∼10 min. Self-cleavage reaction was initiated by adding 5× selection buffer (250 mM HEPES, pH 7.5 at 23°C, 750 mM NaCl, 50 mM MgCl_2_ and 0.005% Tween-20) to the library to produce the final concentrations of 1× selection buffer and 0.2 µM of the library. The reaction was first incubated at room temperature for 2 h. Then, the reaction condition was alternated between room temperature and 37°C every 30 min for another 2 h. The reaction was stopped by adding EDTA (pH 8.0 at 23°C) to a final concentration of 20 mM. The self-cleaved oligonucleotides were separated from the inactive species by 8% denaturing PAGE and used as the templates for PCR amplification.

The oligonucleotides were PCR-amplified in a volume of 50 µL containing 75 mM Tris-HCl (pH 9.0), 2 mM MgCl_2_, 50 mM KCl, 20 mM (NH_4_)_2_SO_4_, 0.2 mM each of the four dNTPs, 1.25 U *Thermus thermophilus* DNA polymerase (Biotools), 0.5 µM DNA primer 1 (5′-TTACA- TCTACAAACATGGTTCGATTCTTGA-3′) and 0.5 µM primer 2 (5′-CGATGCGGGTGCCA-3′). The PCR was carried out for 10 thermocycles of which the temperature was altered in the following order: 94°C, 30 s (2 min for the first cycle); 45°C, 45 s; 72°C, 45 s. A small portion of the PCR products was amplified in the second PCR using a similar condition described above but with 0.3 µM primers 2 and 3 (5′-TTACATCTACAAACATGGTTCGrA-3′) for 15 cycles of 94°C, 30 s (2 min for the first cycle); 53°C, 45 s; 72°C, 45 s. ^32^P-labeling of the oligonucleotides was conducted similarly as in the second PCR but with 30 µM each of the four dNTPs, 10 µCi [α-^32^P]dGTP, 0.2 µM primers 2 and 3 in a total volume of 25 µL. The non-radiolabeled and ^32^P-labeled PCR products were combined, precipitated with ethanol, resuspended in 90 µL of 0.25 M NaOH and incubated at 90°C for 10 min to cleave the single ribonucleotide linkage (rA) within one of the two strands. 10 µL of 3M NaOAc (pH 5.2) was subsequently added to neutralize the solution. The cleaved DNA strand was isolated by 8% denaturing PAGE, eluted from the gel, precipitated with ethanol and restored into the self-cleaving construct by DNA phosphorylation followed by DNA ligation, as described above. The selection was performed for another 11 cycles as described for the first round, except that the final DNA concentration in the self-cleavage reaction was reduced to ∼0.1 µM and the reaction time was progressively shortened to 1 h in round 6, 10 min in round 7, 1 min in rounds 8 and 9, 30 s in round 10 and 6 s in rounds 11 and 12. Population G12 was cloned and sequenced for further analyses.

As we found that G12 was dominated by selfish DNAs, some changes in the selection scheme have been made in order to reduce their chances of survival (see Results section for details). The selection experiment was re-initiated using the G5 population and continued for another 7 cycles, where the incubation time was shortened from 1 min in round 6 (G5) to 8 s in rounds 7 and 8, 5 min in rounds 9 and 10, and 10 s in rounds 11 and 12. G12 was cloned and sequenced for further analyses.

### Reselection

Two partially randomized libraries were chemically synthesized with a degeneracy of 0.3 based on the sequences of truncated MgZ-5 and MgZ-7 (Figure S2). The wild type sequence of MgZ-5 is 5′-TTCTTGATCAACCAGGTCGGGGCCGAAATATAAGATGTTTTGGGAGGCT- AAGCTAGGAAGGACCACCCGCATCG-3′, where the randomized region is underlined; the wild type sequence of MgZ-7 is 5′-TTCTTGATCAAGGATTATTACCAGGTCGGGGCCAAA- TTAACGGAGTTAATTAGGGAGGCTGGCACCCGCATCGTCGGTAGTC-3′. Note that the 3′ primer-binding sites are different from that in the starting library. They were changed by design to avoid cross-contamination by each pool or by the parent population from the initial selection experiment. Consequently, primers 2 for PCR-amplification were revised to 5′-CGATGCGGGTGGTC-3′ and 5′-GACTACCGACGATGC-3′ for MgZ-5 and MgZ-7, respectively. 705 pmol of MgZ-5 sequence variants and 718 pmol of MgZ-7 variants were subjected to 6 rounds of selection based on the original selection scheme described above. The incubation times for rounds 1 to 6 were 30 min, 5 min, 30 s, 10 s, 5 min and 30 s. G6 populations were cloned and sequenced for further analyses.

### Kinetic analyses of the DNA enzymes

Radioactive assays were carried out at 30°C under a single turnover condition with 5 nM substrate (see Text S1 for substrate preparation) and 1 µM DNAzyme in 70 mM HEPES (pH 8.0) containing 40 mM MgCl_2_, 0.001% Tween-20. Reactions were initiated by adding each DNAzyme to a relevant substrate that was pre-incubated in the reaction buffer, and stopped by adding EDTA to a final concentration of 80 mM. The cleavage products were precipitated with ethanol and separated by 10% denaturing PAGE. Cleavage fractions were quantitated by using the Storm 820 phosphorimager along with the Molecular Dynamics software. Cleavage assays were conducted over 10 time points over the course of 1 h or 9 time points over the course of 30 min (in the cases of M18 and M19 constructs; see Figures S7 and S9). Each time point was repeated in at least three independent experiments. Rate constants were obtained by fitting the cleavage fraction vs. time data to either a single (*Y* = *Y*_f_[1−e^−*k*_c_^ *^t^*]) or a double (*Y* = *Y*_f1_[1–e^−*k*_c1_^ *^t^*]+*Y*_f2_[1−e^−*k*_c2_^ *^t^*]) exponential equation with *R* ^2^ >0.99 by using GraphPad software Prism 4.03. *Y* represents cleavage yield; *Y*_f_ represents final cleavage yield; *k*_c_ represents the observed cleavage rate.

Fluorescent assays were performed on a Cary Eclipse Fluorescence Spectrophotometer (Varian). Fluorescence signals were recorded every 15 s at 800 V. Fluorescein was excited at 495 nm (5 nm bandwidth) and fluorescence was monitored at 520 nm (5 nm bandpass). Fluorescence of the substrate in the reaction buffer was monitored for at least 5 min before the addition of the DNAzyme. Fluorescence after DNAzyme addition was monitored for at least 30 min. Background signal was determined from the fluorescence output of the reaction buffer alone. The average background signal over the course of at least 5 min of fluorescence monitoring was subtracted from the sample readings before *F*/*F*_o_ was computed. Thus, *F*/*F*_o_ = (*F*−average background)/(average *F*_o_−average background). *F* represents fluorescence reading; average *F*_o_ is the average signal of the substrate during the initial ∼5 min of fluorescence monitoring; *F*/*F*_o_ represents signal enhancement. The final reaction condition was the same as described above for the radioactive assays. Fluorescence kinetics for every DNAzyme construct was done in triplicate. The signaling rates were determined by fitting the *F*/*F_o_* vs. time data to a modified single exponential equation (*F*/*F*_o_ = initial *F*/*F*_o_+(final *F*/*F*_o_−initial *F*/*F*_o_)×(1−e^−*k*_s_^ *^t^*); initial *F*/*F*_o_ = 1.0) with *R* ^2^>0.93. *k*_s_ represents the rate of signal enhancement.

### Assays to test sensor performance

Radioactive cleavage assays were carried out at room temperature with 5 nM substrate-aptamer complex and 50 nM (or 1 µM in the case of ADP sensor) DNAzyme in 50 mM HEPES (pH 7.0) containing 20 mM MgCl_2_, 0.001% Tween-20. Note: these reaction conditions represent the optimal conditions that yield the best signal enhancement in the presence of 1 mM target. Reactions were initiated by adding the DNAzyme to the substrate-aptamer complex that was pre-incubated in the reaction buffer containing the ligand at various concentrations. Each reaction was stopped by adding EDTA to a final concentration of 40 mM. The cleavage products were precipitated with ethanol and separated by 10% denaturing PAGE. Cleavage fractions were quantitated by phosphorimaging.

Fluorescent assays were performed similarly. The fluorescence intensity of the substrate-aptamer complex in the absence or presence of each relevant target was monitored for at least 5 min before the addition of the DNAzyme. Fluorescence was monitored for ∼90 min (for ATP sensor) or ∼60 min (for ADP sensor) after the DNAzyme addition. Signals were recorded every 30 s. *F*/*F*_o_ was computed as described above. Note that *F*_o_ here is the signal of the substrate-aptamer complex with or without the ligand present at various concentrations before the addition of the DNAzyme. Fluorescence kinetics for every ligand type or concentration was done in triplicate. The initial rate of signal enhancement (*v*_o_) was approximated by using a linear equation (*F*/*F*_o_ = *v*_o_ *t*+1.0; *F*/*F*_o_ = 1.0 at *t* = 0 min) to fit the *F*/*F_o_* vs. time data, which were collected during the first ∼10 min (for ATP sensor) or ∼3 min (for ADP sensor) once DNAzyme was added to the reaction mixture. The dissociation constant of the ligand (*K*_d_) was extracted by fitting the *v*_o_ vs. [ligand] data to the one site binding hyperbola equation (*v*_o_ = (*v*_max_×[ligand])/(*K*_d_+[ligand])) with *R* ^2^ >0.98 (note: *v*_max_ = maximal rate of signal enhancement; [ligand] = ligand concentration).

## Supporting Information

Text S1 Supplementary Methods and Figure Legends
(0.05 MB DOC)

Figure S1 MgZ species and selfish DNAs from G12
(3.38 MB TIF)

Figure S2 Selfish DNA G12SD-1
(0.95 MB TIF)

Figure S3 Mutational analysis of MgZ-5 through reselection
(0.43 MB TIF)

Figure S4 DMS methylation interference pattern of a selected MgZ
(1.56 MB TIF)

Figure S5 Initial truncation study of MgZ
(1.72 MB TIF)

Figure S6 Characterization of stem P3
(1.70 MB TIF)

Figure S7 Deletion study of P1 and P2
(2.41 MB TIF)

Figure S8 Substrate flexibility
(1.50 MB TIF)

Figure S9 Kinetic analyses of various DNAzyme constructs coupled with S3 and S4
(0.63 MB TIF)

Figure S10 pH effects of nucleic acid analogs
(3.21 MB TIF)
