# Supplementary material for: Simple Fluorescent Sensors Engineered with Catalytic DNA ‘MgZ’ Based on a Non-Classic Allosteric Design
Source: PLoS One. 2007 Nov 21;2(11):e1224. doi: 10.1371/journal.pone.0001224 (PMC2077808; doi:10.1371/journal.pone.0001224)
Supplement: Text S1 — Supplementary Methods and Figure Legends (0.05 MB DOC) [file pone.0001224.s001.doc]

**Text S1**

**Supplementary Methods**

**DMS footprinting.** The protocol described here is similar to that used in our previous studies [1]. In brief, 80 pmol of the non-radiolabeled and self-cleaving DNAzyme (Figure 1) were allowed to self-cleave in 1× selection buffer in a total volume of 400 μL for 10 min. The oligonucleotides were then ethanol precipitated, resuspended in 400 μL of H_2_O, heated at 90^o^C for 30 s and cooled to room temperature for 10 min. 400 μL of 0.4% DMS in H_2_O was added, and the mixture was incubated at room temperature for 35 min. The resulting methylated oligonucleotides were denoted as the “control”. A similar procedure but performed in the opposite order (methylation first, self-cleavage afterwards) was applied to another 80 pmol of the DNAzyme. These oligonucleotides were denoted as the “test”. Both the control and test oligonucleotides were recovered by ethanol precipitation and ^32^P-labeled at the 5'-ends by T4 PNK. The longer cleavage fragment was isolated by 10% denaturing PAGE. The purified DNA was resuspended in 50 μL of H_2_O and mixed with 50 μL of 20% piperidine. The mixture was incubated at 90^o^C for 30 min and subsequently dried under vacuum. The DNA fragments were separated by 15% denaturing PAGE. A phosphorimage of the gel was obtained, and the band intensities of correlated fragments (control and test) were quantitated and compared using the Molecular Dynamics software.

**Substrate preparation.** Substrate S1 was made by first ^32^P-labeling the 5'-end of the 3'-tail oligonucleotide, 5'-TTCTTGATCAA-3', using T4 PNK. The 3'-tail was then annealed to the chimeric DNA/RNA oligonucleotide, S2 (5'-GATGTGTCCGTGCFrAQGGTTCGA-3'), using a DNA splint and T4 DNA ligase. The ligated product was purified by 10% denaturing PAGE. Substrate S3M was prepared similarly by ligating a different 3'-tail (5'-CTCATCCGT-3') to another chimeric oligonucleotide (5'-GATGTGTCCGTGCFrAQG-3'). S4 and S5M were made by ligating the central chimeric oligonucleotide, 5'-GCFrAQGGTT-3', to a specific 5'-end oligonucleotide and the respective 3'-tail. See Figures S7 and S8 for the entire sequences. Note that the 3'-tails are the only oligonucleotides that were ^32^P-labeled in the substrate constructs above. S2 and S3 were ^32^P-labeled at the 5' ends.

**Secondary structure characterization.** Cleavage activities of various DNAzyme constructs were assessed in trans, using several substrates with different sequence contexts (Figures S5-S8). Cleavage reactions were initiated by adding DNAzyme to a particular substrate, which was pre-incubated in the reaction buffer. The final concentration of each constituent in the reaction mixture was 5 nM substrate, 250 nM DNAzyme, 1 × selection buffer. The reactions were stopped after 5 or 10 min of incubation by adding EDTA to 20 mM. The oligonucleotides were ethanol precipitated before analyses by 10% denaturing PAGE.

**Supplementary Figure Legends**

**Figure S1.** MgZ species and selfish DNAs from G12. The name fixed on the left of each sequence designates the sequence class and clone number. All MgZ species were confirmed to utilize Mg^2+^ for catalysis. Selfish DNAs are denoted with “SD”. G12SD oligonucleotides were isolated via the original selection scheme. TMESD oligonucleotides are DNA species that survived the modified selection scheme, which involves TME-based denaturing PAGE. Scale at the top corresponds to the nucleotide positions between the two PCR primer-binding sites in PCR 1 (Figure 1 inset).

**Figure S2.** Selfish DNA G12SD-1. G12SD-1 was subjected to reselection for sequence optimization. Seven selection cycles were performed, and the final generation was cloned and sequenced. Clone #13 (5'-CTGGCCTAAATGGGGAAAAAAAAGAATCGAAGGGTTTGAGGTTCGGTGGGTAGCACGGA -3'; only random region is shown) was arbitrarily chosen for further examination. (A) Intramolecular cleavage assay. The ribo-linkage within the self-cleaving construct of clone #13 was already cleaved during its making (lane 1, clone #13 in H_2_O). We suspected that the non Mg^2+^-dependent self-cleavage occurred when the oligonucleotides were being recovered from the denaturing PAGE gel. Further incubation in 1 × selection buffer for 10 s, 1 min and 5 min (lanes 2-4) did not yield extra cleavage products. ►, precursor; ▷, 3' cleavage fragment. (B) Intermolecular cleavage assay. Reaction condition: 50 nM substrate (5'-GATGTGTCCGTGCFrAQGGTTCGATCCGAGAATT-3'), 5 µM DNAzyme, 50 mM HEPES, pH 7.5, room temperature, 10 mM MgCl_2_ (if added). NaCl in lanes 4-8: 100, 200, 300, 400, 500 mM. Reaction was initiated by adding DNA enzyme to the substrate, and stopped by adding ethanol after 5 min of incubation. Note: higher [MgCl_2_] (up to 100 mM) had no effect on cleavage yield (data not shown).

**Figure S3.** Mutational analysis of MgZ-5 through reselection. Six rounds of selection were performed, and a total of 56 clones were sequenced. By using these 56 sequences, a variation index for each nucleotide position within the randomized region was computed. Variation index = observed % mutation / % mutation of the starting library. Shown under the chart is the MgZ-5 sequence coded with different colors at each nucleotide position. Black, absolutely conserved; cyan, variation index between 0 and 0.5; green, 0.5-1.0; red, >1.0. Constant regions (chimeric substrate, 5' and 3' primer-binding sites) are shown as “▬”.

**Figure S4.** DMS methylation interference pattern of a selected MgZ. DNA bands are read from the bottom to the top in the 5'→3' direction. The bands are assigned with the respective regions on the left according to the structural model in Figure 1B. C represents the control (methylation after self-cleavage); T, the test (methylation before self-cleavage). Reduced intensities of certain bands in lane T relative to those in lane C indicate that the methylation at N7 of guanine or N3 of adenine interfered with the catalytic activity of MgZ. These bands are denoted with arrows and the corresponding nucleotides. Note: Only nucleotide positions that have a ratio of band intensity in lane C over the band intensity in lane T >4:1 are considered to be intolerable to methylation. Interestingly, there are several bands that show converse intensity pattern (boxed), which might implicate enhancement in cleavage activity upon methylation at the concerned adenines. See Supplementary Methods for the protocol of DMS

footprinting assay.

**Figure S5.** Initial truncation study of MgZ. (A) A secondary structure model of MgZ in the trans format. MgZ-WT (WT = wild type) comprises all the nucleotides in the self-cleaving construct shown in Figure 1B, except the substrate portion. S1, substrate 1. (B) Truncations and mutations in stem P2 and stem loop P3-L3. Specific modifications made in MgZ-WT are shown in various mutant constructs (MX, X = 1, 2, 3…). (C) Cleavage activities of MXs shown in panel B were assessed by 5 min cleavage assays in 1 × selection buffer using S1 or S2. % clv, % in cleavage yield; ND, not detectable. ►, substrate; ▷, one of the cleavage fragments. There is only one cleavage fragment shown in each lane as the other cleavage fragment was non-radiolabeled and thus not revealed by phosphorimaging. See Supplementary Methods for details in substrate making. Lane 1 is the negative control for S1, where no DNAzyme was added to the reaction mixture. Comparing with lane 2, lane 3 shows the G•T to A=T change in P3 tremendously reduced the cleavage activity of MgZ while lane 4 shows a deletion of 3 nucleotides at the very 5' end had no effect on catalysis. Lane 5 is the negative control for S2. Lanes 6-8 indicate that S2 is a better substrate, and further deletion/mutation at the 5' end of the DNAzyme enhanced the cleavage activity. Inset shows the substrate sequences.

**Figure S6.** Characterization of stem P3. (A) A secondary structure model of S2/MgZ-M5 complex. M5 is a mutant similar to M3 (Figure S5B), except that its 3' end was modified to interact with S2 in a full Watson-Crick duplex. (B) Truncations and mutations in P3. Dotted brackets highlight the changes with reference to M5. Brackets with branched lines highlight the changes with reference to M10. (C) 5 min cleavage assays were carried out using S2 and various mutant constructs shown in panel B. Lane 1 is S2 in 1 × selection buffer with no DNAzyme. Lanes 2-6 show that 1) P3 could be shortened to 5 base pairs although the cleavage activity was compromised; 2) the content of P3, excluding the 3 base pairs closest to J2/3 or J3/1, can be covaried; 3) the content of L3 is not important to catalysis. In conjunction with the previous result in Figure S5C (lane 3), lane 7 here suggests that a G•T wobble pair rather than a Watson-Crick base pair at the third position from the open end is preferred for catalysis. Lanes 8-11 indicate that the absolutely conserved C_46_C_47_ and G_65_G_66_ dinucleotide sequences (Figure 1B), which potentially form 2 C≡G base pairs with each other, cannot tolerate any covariations or mismatches. The formation (and perhaps orientation) of these 2 base pairs requires some structural support in the form of a stem or a stem loop structure (lane 12).

**Figure S7.** Deletion study of P1 and P2. (A) Mutations in P1. M16 and M17 were designed based on M4. The pairing of S2 with M4 has been shown to give the best cleavage yield up to this point (Figure S5C, lane 8). To explore if the catalytic efficiency can be improved by changing the substrate sequence in such a way that it forms a full duplex with M4 at P1, a new substrate, S3, was synthesized. See inset for the entire sequences of the substrates used in this figure. Mutations with reference to S2 are underlined. (B) 5 min cleavage assays were carried out using the substrate/mutant pairings shown in panel A. Lanes 2 and 4 suggest that a full duplex at P1 does not provide any extra thermodynamic stability to the folding of the DNAzyme, as S2 and S3 yielded a similar amount of cleavage products. However, when the full duplex configuration was imposed by altering the sequence of the DNAzyme instead of the substrate, a considerably lower cleavage yield was resulted (Figure S6C, lane 2). This implies that MgZ is susceptible to modification at the 3' substrate-binding arm, probably because alternative folding(s), which are catalytically inactive, might be formed. Given that S3 pairing with MgZ has a simpler secondary structure, S3 was employed for the truncation study of P1. As indicated in lanes 5 and 6, P1 can be shortened to 6 base pairs without a drop in cleavage activity. (C) Truncation of P1 and P2 using S4. S4 is essentially identical to S3, except that 1) there are 2 extra purines at the 3' end (see inset); 2) S4 is a better substrate in terms of its cleavability (Figure S9A); 3) S4 is able to generate a larger *F*/*F*_o_ (Figure S9B). M18-M22 were designed based on M17. (D) 5 min cleavage assays were carried out using the substrate/mutant pairings shown in panel C. Lanes 2-5 indicate that 5 base pairs in P2 are optimal for activity, while lanes 2, 6 and 7 show that a minimum of 6 base pairs at P1 are required for optimal activity.

**Figure S8.** Substrate flexibility. (A) S3/M8 is a reference pairing for comparison. S5M was designed to carry mutations on the 5' of S3. S3M, on the other hand, carries mutations on the 3'. M23 was designed to interact with S5M with perfect Watson-Crick base pairs, while M24 was designed to interact with S3M optimally. Inset shows the substrate sequences. (B) 5 or 10 min cleavage assays were carried out using the substrate/mutant pairings shown in panel A. Substrate/mutant pairings that underwent 10 min of cleavage are highlighted with “*” behind the name of the mutant. Lanes 2-4 and 10-11 suggest that 1) MgZ does not rely heavily on its 3' end for substrate-binding; 2) P1 could be as short as 2 nucleotides as long as the 3' substrate-binding arm remains unchanged (lanes 2 and 11); 3) P1 can be covaried but the cleavage activity would be compromised (lanes 3, 4 and 10). Lanes 6-8, 10 and 12 indicate that 1) MgZ is very sensitive to mismatches in P2 (lanes 6 and 12); 2) Similar to P1, P2 can be covaried but with a significant

drop in cleavage activity (lanes 7, 8 and 10).

**Figure S9.** Kinetic analyses of various DNAzyme constructs coupled with S3 and S4. (A) Cleavage kinetics. Cleavage reactions were carried out with 5 nM S3 (- - -) or S4 (──) and 1µM DNAzyme in 70 mM HEPES, pH 8.0, 40 mM MgCl_2_, 0.001% Tween-20 at 30^o^C. Error bar represents the standard deviation of three independent assays. The % cleavage versus time data of S4/M17, S4/M18 and S4/M19 pairings were fitted to a single exponential equation (*Y* = *Y*_f_[1 – e^-^*^k^*_c_*^t^*]) with *R*^2^ >0.99. In contrast, the data for S3/M4 and S3/M17 were fitted to a double exponential equation (*Y* = *Y*_f1_[1 – e^-^*^k^*_c1_*^t^*] + *Y*_f2_[1 – e^-^*^k^*_c2_*^t^*]) with *R*^2^ >0.99. Inset: Differences in the final cleavage yield are clearly shown. (B) Fluorescence kinetics. Fluorescence-monitored cleavage assays were conducted similarly as the radioactive assays. The *F*/*F*_o_ versus time data were fitted to a modified single exponential equation (*F*/*F*_o_ = initial *F*/*F*_o_ + (final *F*/*F*_o_ – initial *F*/*F*_o_) × (1 – e^-^*^k^*_s_*^t^*); initial *F*/*F*_o_ = 1.0) with *R*^2^ >0.93. Conclusion from panels A and B: S4/M17 is the best combination for fluorescence-signaling purposes.

**Figure S10.** pH effects of nucleic acid analogs. (A) Nucleic acid analogs have no effect on MgZ activity. S4 was preincubated with 1 mM nucleic acid analog (unless specifically stated otherwise) in 50 mM HEPES, pH 7.0, 20 mM MgCl_2_, 0.001% Tween-20 at room temperature. Cleavage reaction was initiated by adding MgZ-M17 and stopped by adding EDTA to a final concentration of 40 mM in 5, 10 or 20 min. The most left lane in each sub-panel is the reaction mixture without the DNAzyme. Although the cleavage kinetics seems to be affected by the presence of 2 mM ATP, the cleavage yield in >10 min of incubation is commensurate with that in the absence of any ligand. (B) Adenosine and AMP have no effect on fluorescence signals. Cleavage of S4 in the absence (open symbol) or presence (filled symbol) of 1 mM adenosine or AMP was initiated by adding the DNAzyme. 5 µL of 10 mM nucleic acid analog (open symbol) or H_2_O (filled symbol) was then added to the reaction mixture (50 µL) after ~28 min of fluorescence monitoring. This addition is highlighted by an arrow. The final reaction condition was similar to that in the radioactive assays. To clarify the labeling system used in this figure, “H_2_O/adenosine” would be used as an example. “H_2_O/adenosine” refers to that the cleavage reaction was first conducted in the absence of any ligand. After a certain period of fluorescence monitoring, ~1 mM adenosine was then included in the reaction. F.U., fluorescence unit. (C) ADP and ATP quench fluorescence. Fluorescence-monitored cleavage assays were carried out similarly as described in panel B. The final concentration of ADP or ATP in the reaction mixture was ~1 mM, unless specifically stated otherwise. Since the final fluorescence outputs were not affected by the order of nucleic acid analog or H_2_O addition, fluorescence quenchings were simply caused by the presence of ADP or ATP. (D) Fluorescence rescue with excess buffering agent. Fluorescence assays concerning the effects of 1 mM or 2 mM ATP were repeated here, but with an increase in the concentration of HEPES (pH 7.0) to 150 mM. In comparison with the results in panel C, fluorescence signals in the presence of ATP were recovered to the normal level by having excess buffering agent. This suggests that the addition of a high concentration of ADP or ATP to the reaction mixture could cause a significant drop in pH due to the insufficient buffering capacity in the original reaction condition. This in turn converted the dianionic form of fluorescein to the monanionic form which has a much lower quantum yield. Q.Y. of dianion and monanion = 0.93 and 0.37; the protolytic constant of the equilibrium = 6.43[2].

**Supplementary References**

1. Chiuman W, Li Y (2006) Evolution of High-Branching Deoxyribozymes from a Catalytic DNA with

a Three-Way Junction. Chem Biol 13: 1061-1069.

2. Sjöback R, Nygren J, Kubista M (1995) Absorption and fluorescence properties of fluorescein.

Spectrochim Acta A 51: L7-L21.
