# Supplementary figures and images for: Simple Fluorescent Sensors Engineered with Catalytic DNA ‘MgZ’ Based on a Non-Classic Allosteric Design
Source: PLoS One. 2007 Nov 21;2(11):e1224. doi: 10.1371/journal.pone.0001224 (PMC2077808; doi:10.1371/journal.pone.0001224)

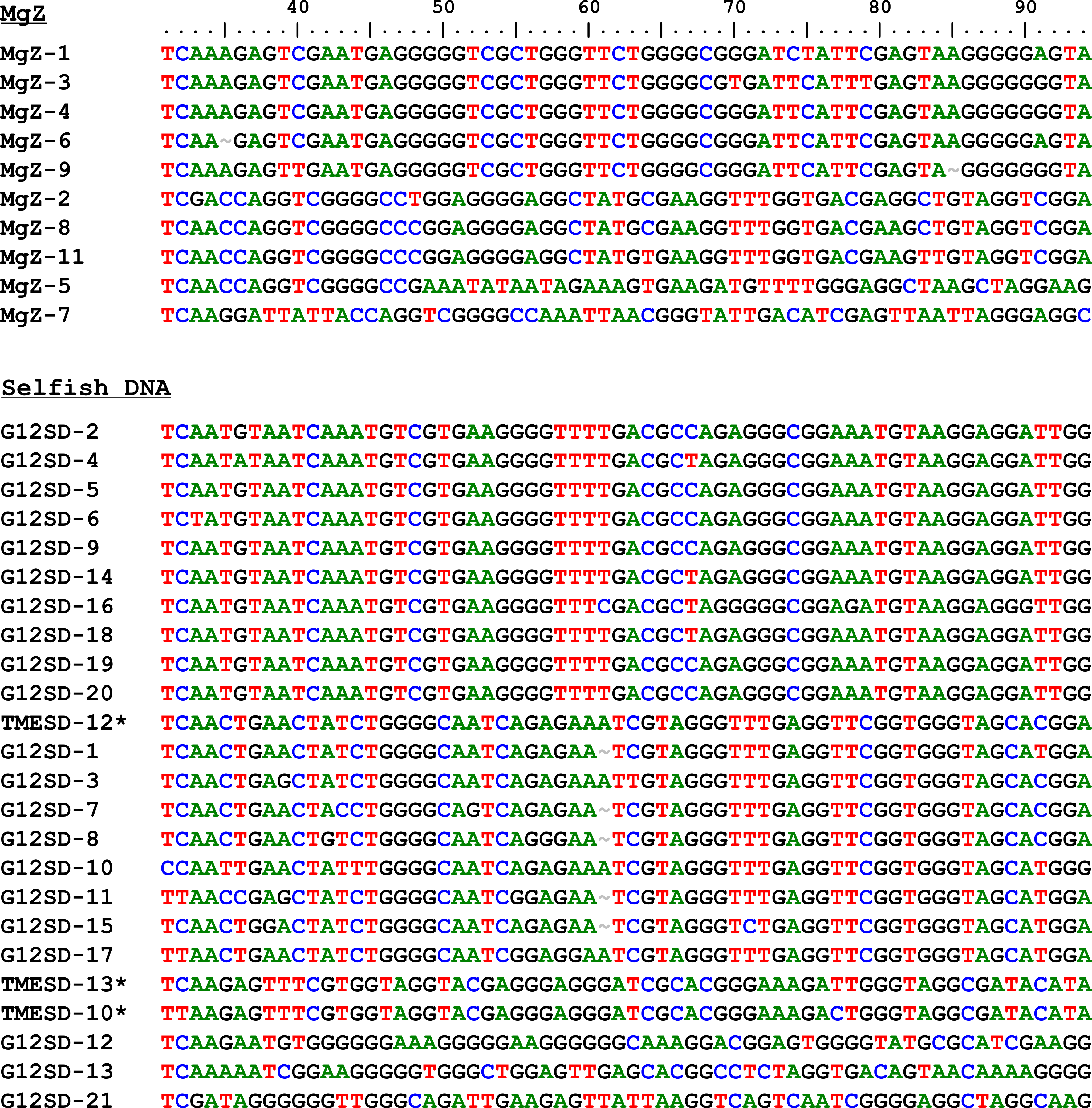

Supplement: Figure S1 — MgZ species and selfish DNAs from G12 (3.38 MB TIF) [file pone.0001224.s002.tif]

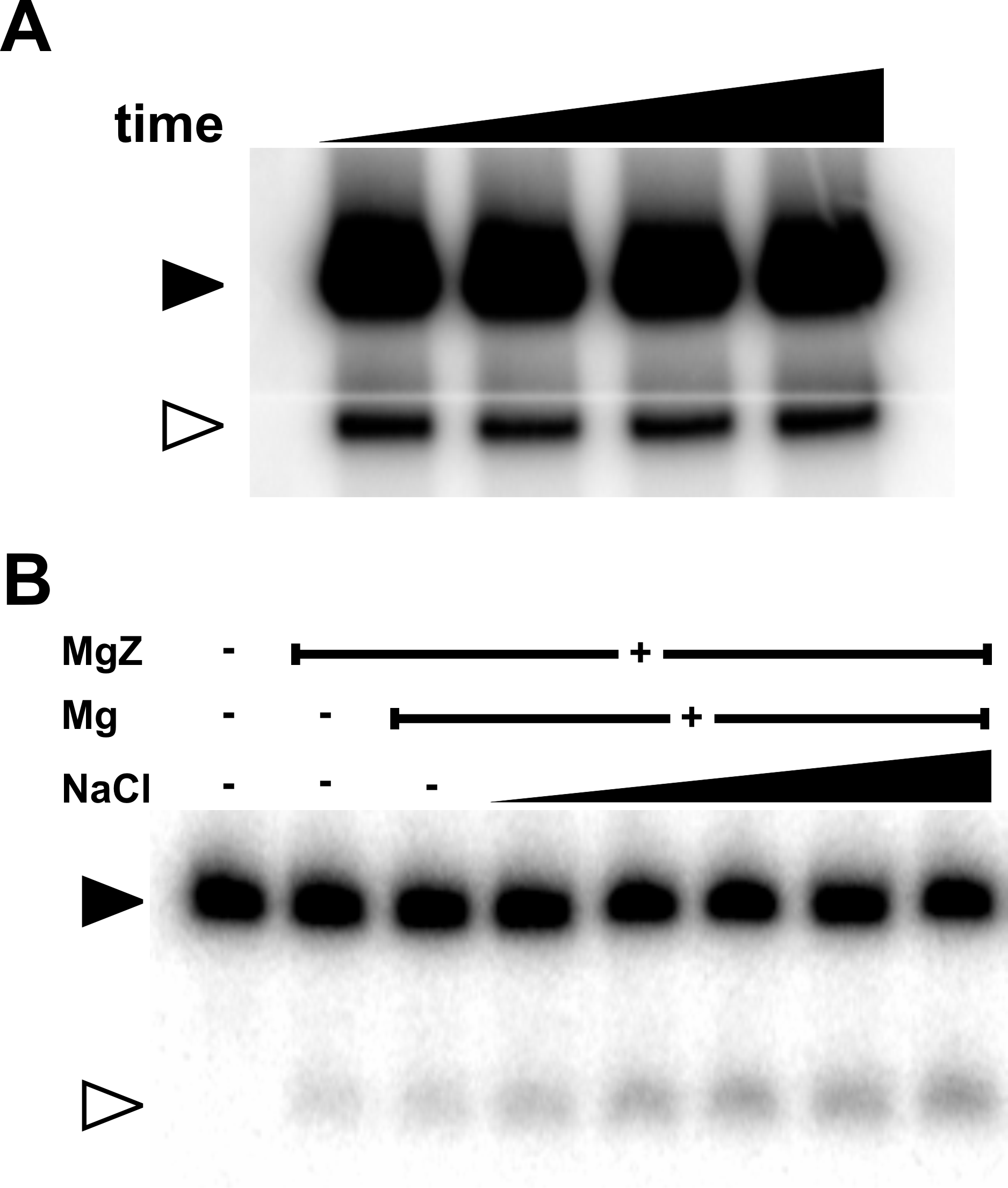

Supplement: Figure S2 — Selfish DNA G12SD-1 (0.95 MB TIF) [file pone.0001224.s003.tif]

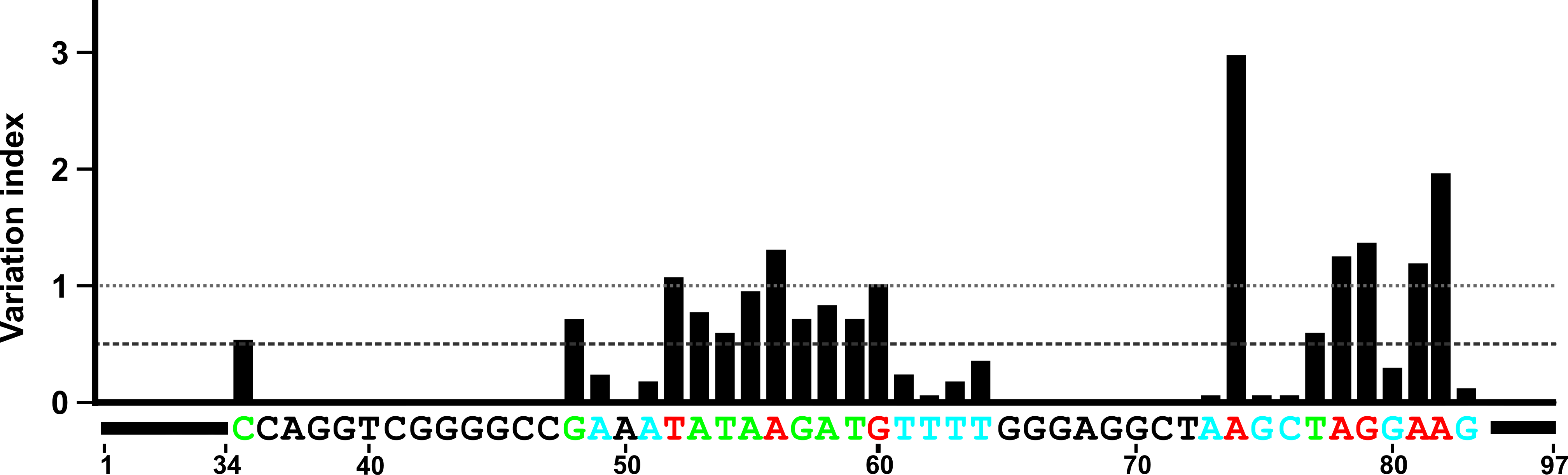

Supplement: Figure S3 — Mutational analysis of MgZ-5 through reselection (0.43 MB TIF) [file pone.0001224.s004.tif]

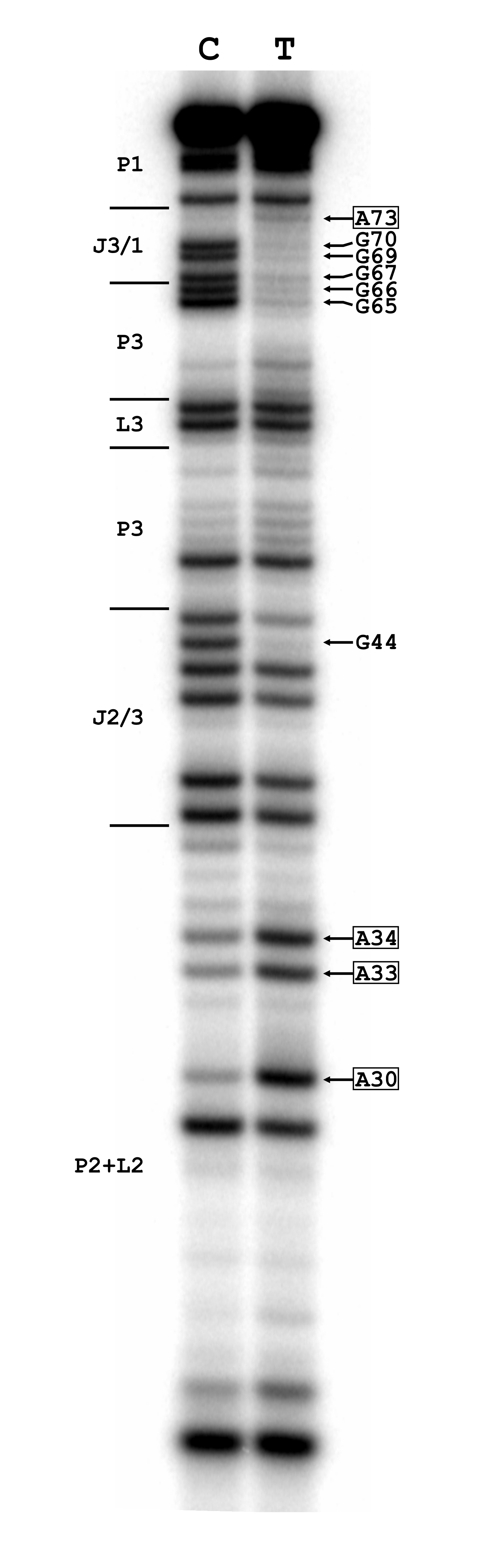

Supplement: Figure S4 — DMS methylation interference pattern of a selected MgZ (1.56 MB TIF) [file pone.0001224.s005.tif]

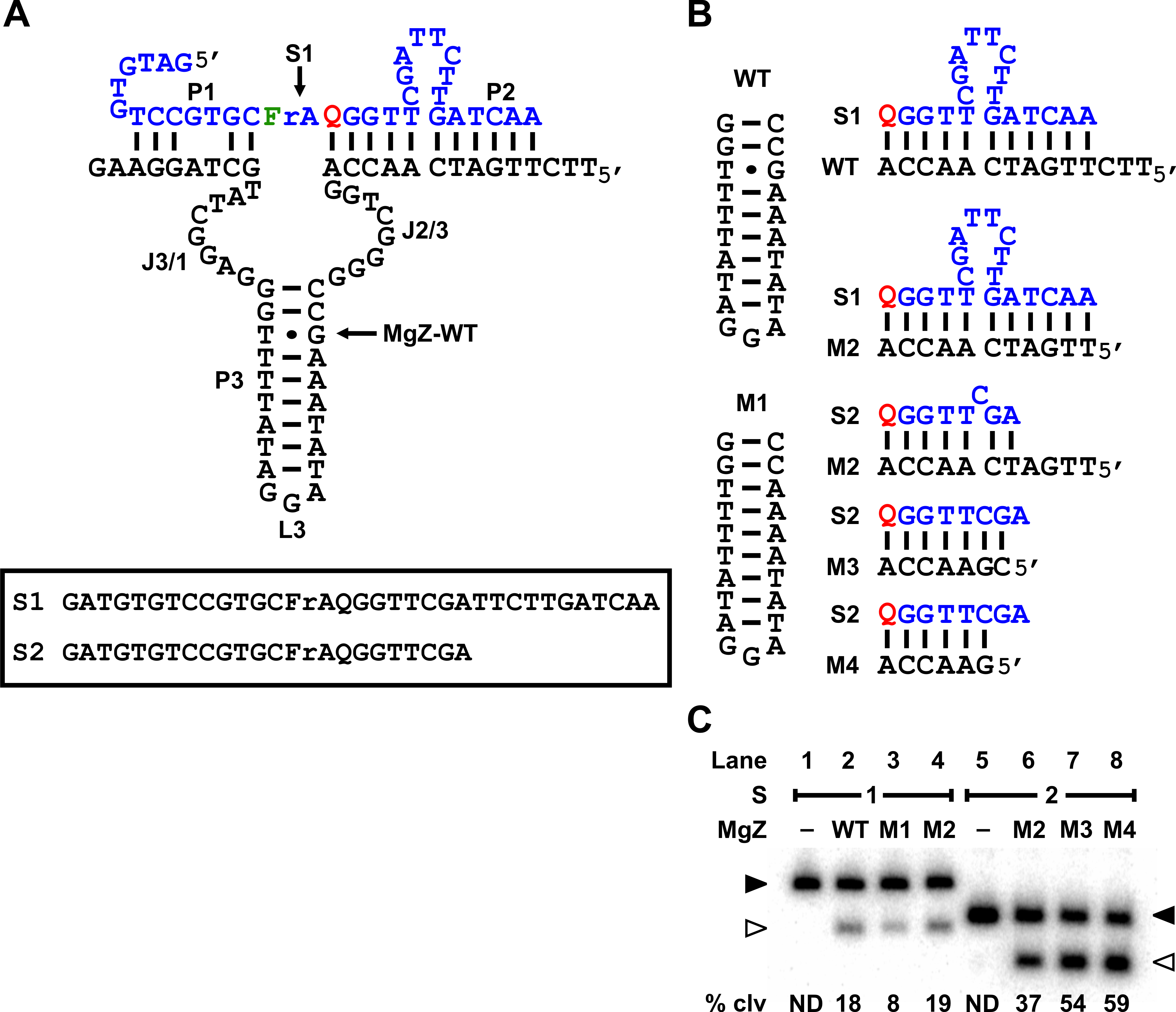

Supplement: Figure S5 — Initial truncation study of MgZ (1.72 MB TIF) [file pone.0001224.s006.tif]

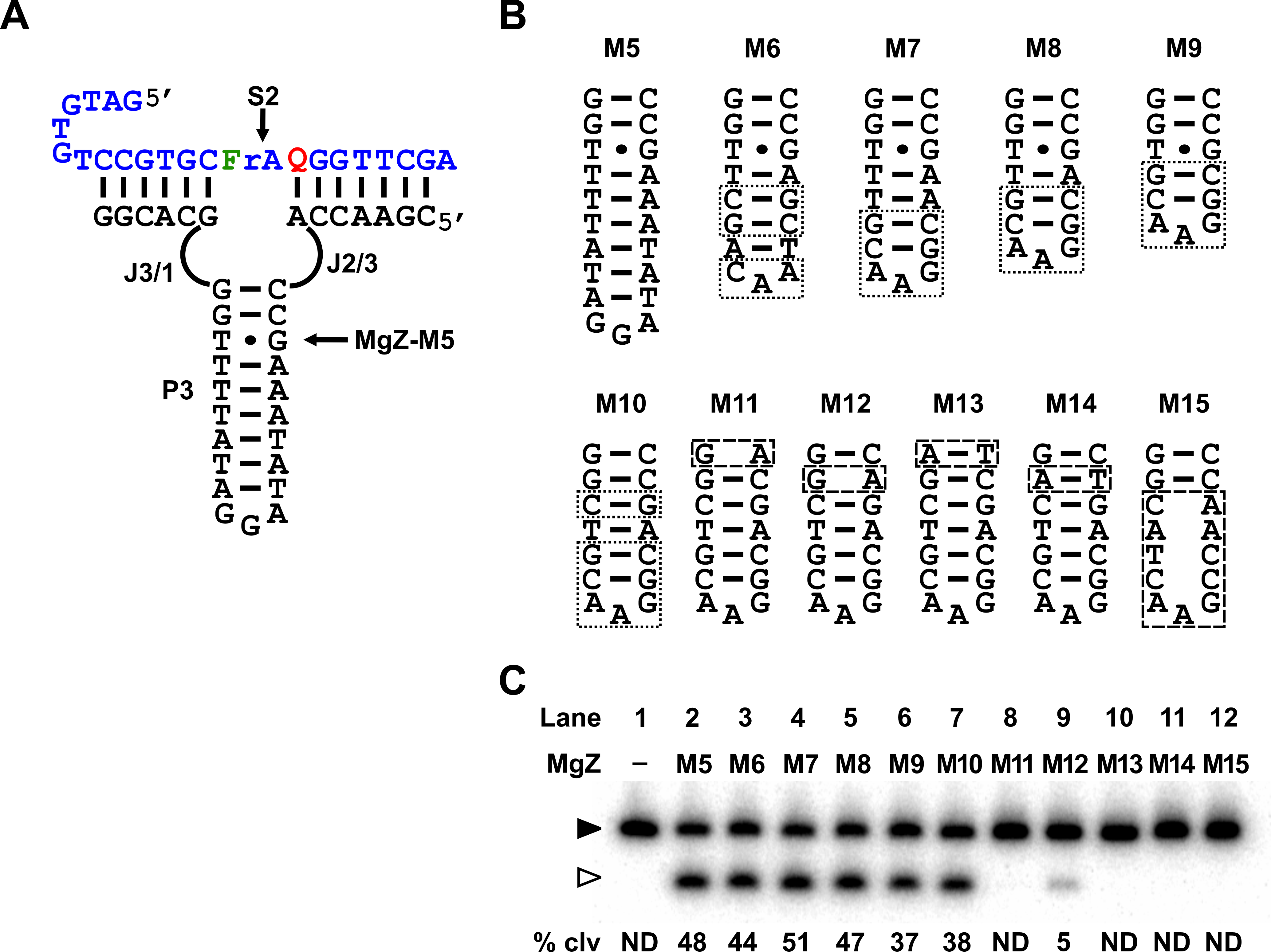

Supplement: Figure S6 — Characterization of stem P3 (1.70 MB TIF) [file pone.0001224.s007.tif]

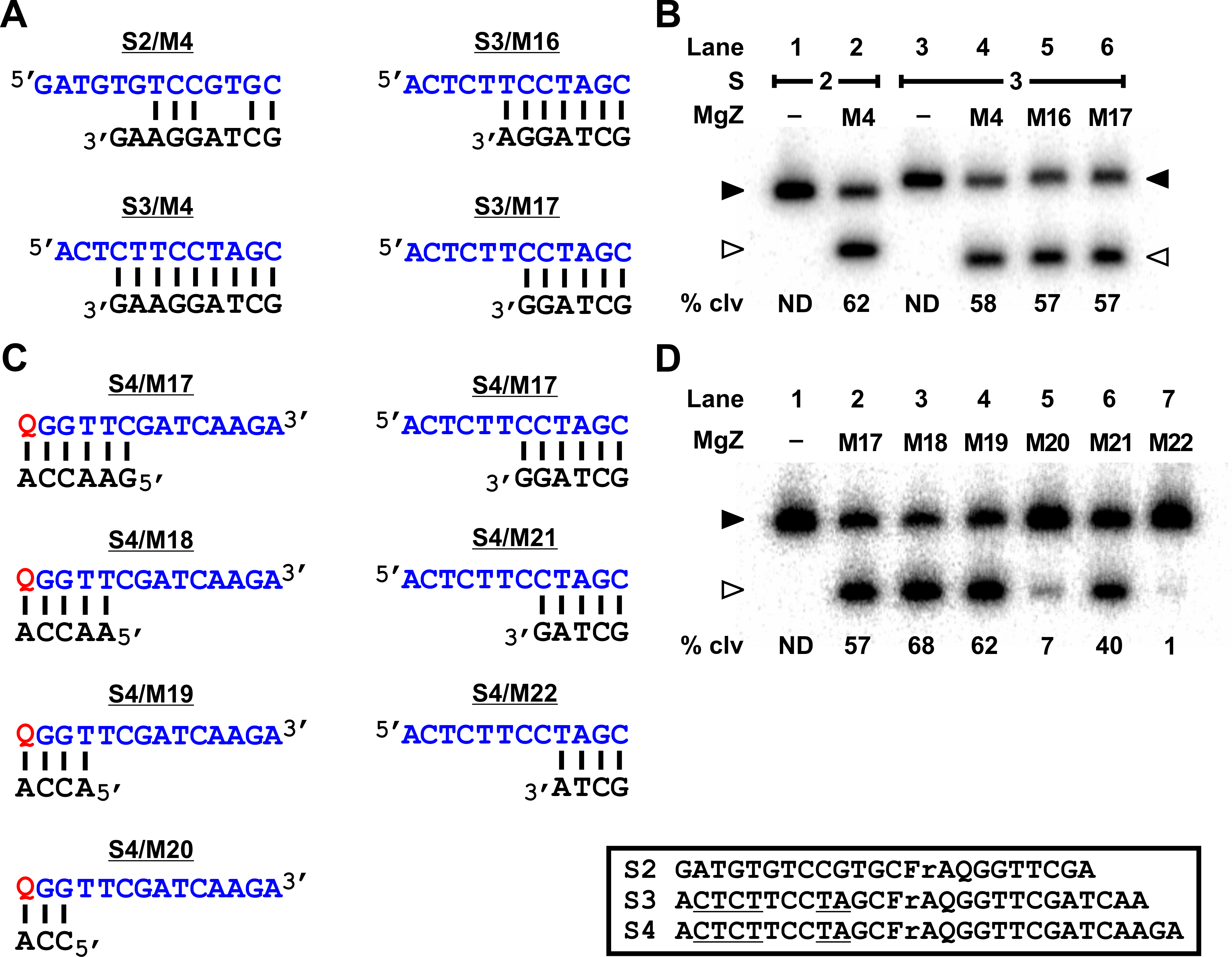

Supplement: Figure S7 — Deletion study of P1 and P2 (2.41 MB TIF) [file pone.0001224.s008.tif]

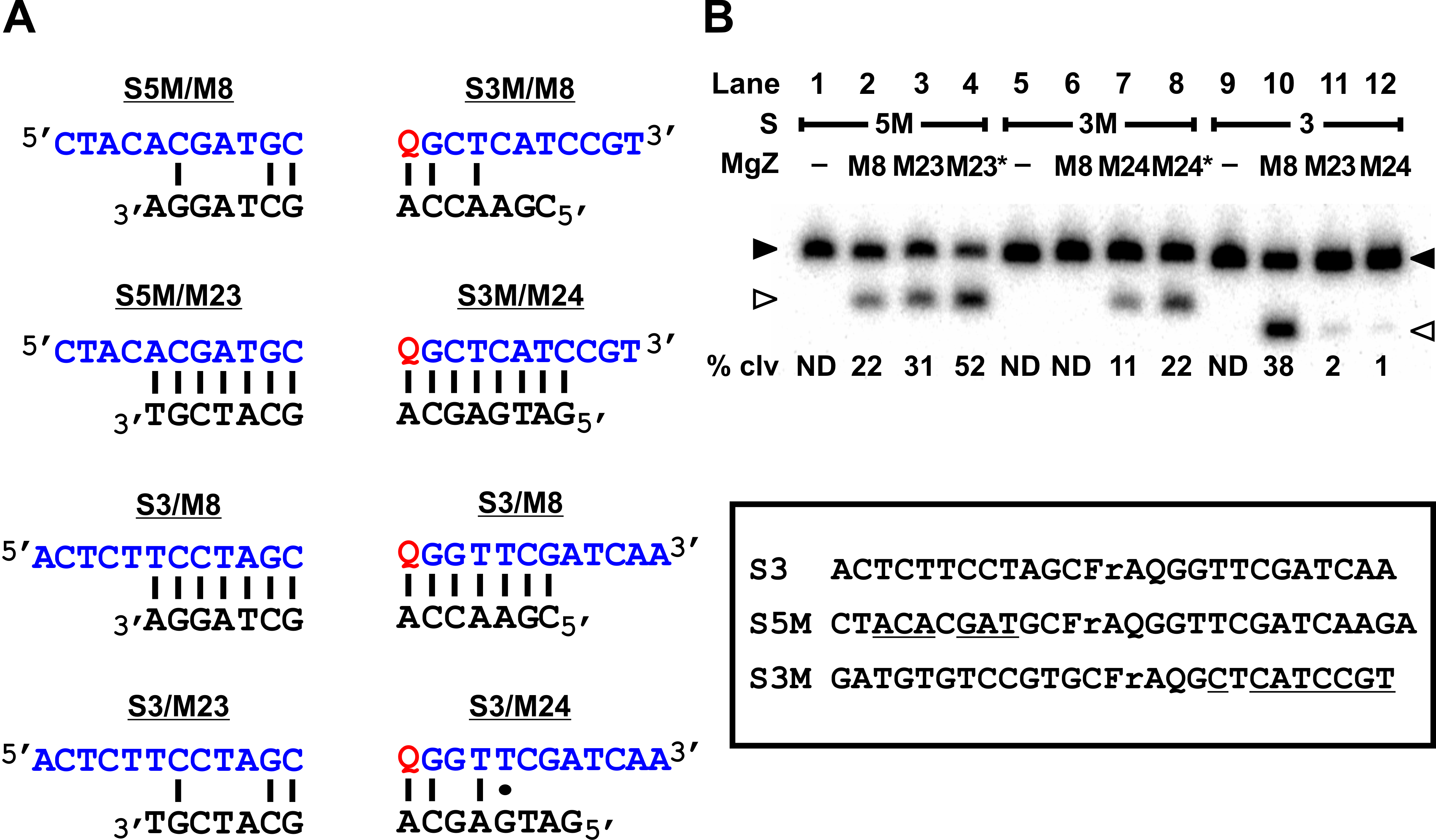

Supplement: Figure S8 — Substrate flexibility (1.50 MB TIF) [file pone.0001224.s009.tif]

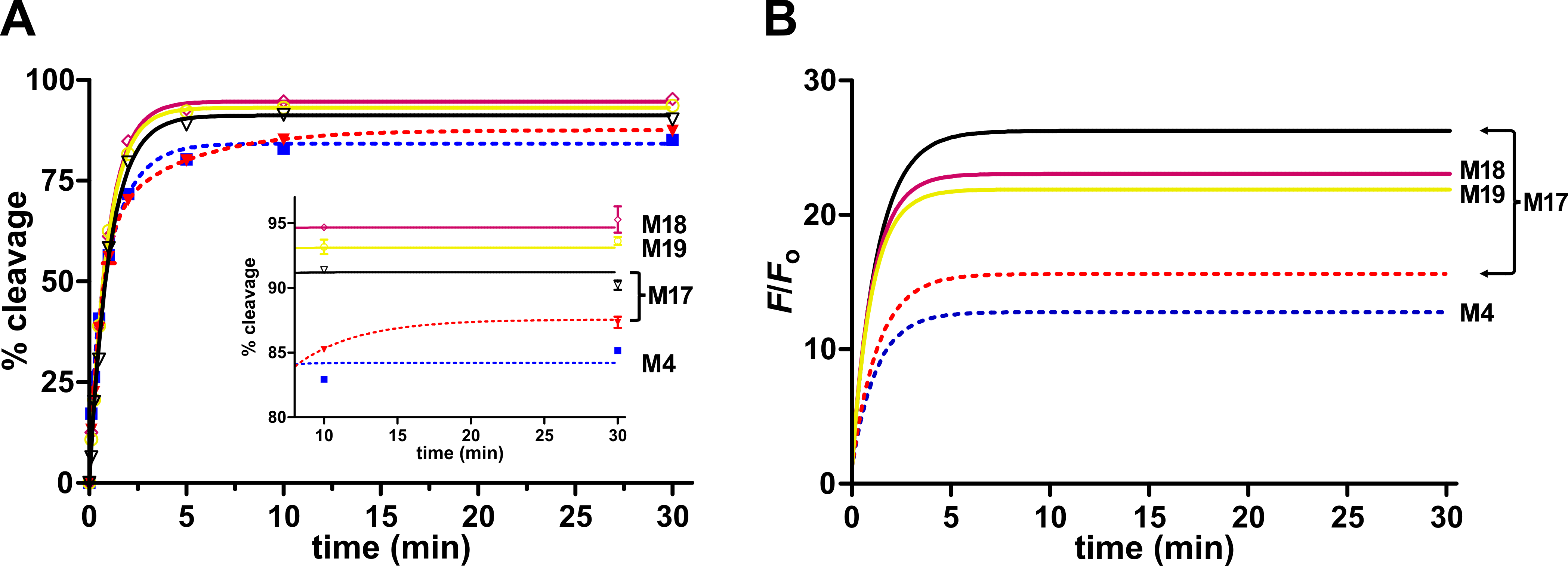

Supplement: Figure S9 — Kinetic analyses of various DNAzyme constructs coupled with S3 and S4 (0.63 MB TIF) [file pone.0001224.s010.tif]

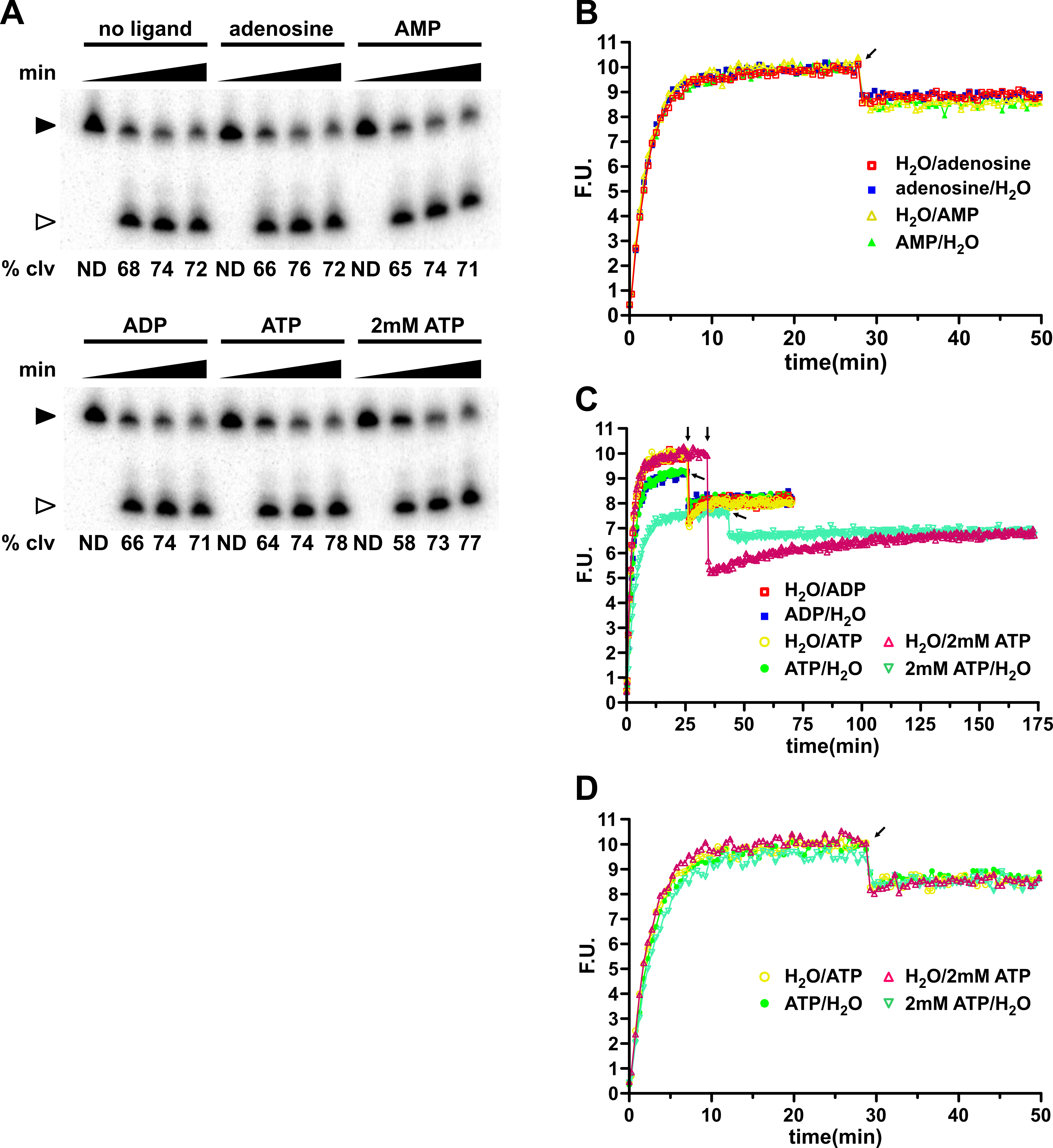

Supplement: Figure S10 — pH effects of nucleic acid analogs (3.21 MB TIF) [file pone.0001224.s011.tif]
